# Estimating recombination fraction via Pearson correlation

**DOI:** 10.1007/s00122-026-05178-w

**Published:** 2026-02-14

**Authors:** Chin-Sheng Teng, Shizhong Xu

**Affiliations:** 1 Department of Statistics, University of California, Riverside, CA 92521 USA; 2 Department of Botany and Plant Sciences, University of California, Riverside, CA 92521 USA

## Abstract

Estimating recombination fractions is crucial for constructing genetic linkage maps and understanding the inheritance patterns of crop genome in breeding populations. Traditional methods, such as the maximum likelihood method, rely on iterative algorithms to estimate recombination fractions in $F_{2}$ populations, which can be computationally intensive. While most existing methods focus on recombination fractions in $F_{2}$, recombination fractions in later generations ($F_{t}$ for $t > 2$) is also important for capturing the increasing resolution of genetic maps over generations. In this study, we introduced a Pearson correlation method for estimating recombination fractions in $F_{t}$ for $t \ge 2$. This is the first study to demonstrate that the Pearson correlation between marker alleles of different loci can be effectively used to estimate the recombination fractions between markers in advanced generations. This method is straightforward, allowing researchers to quickly and efficiently compute recombination fractions, offering a significant speed advantage without compromising estimating accuracy. We evaluated the performance of the new method by comparing it with the expectation–maximization (EM) algorithm across $F_{2}$, $F_{3}$, and $F_{4}$ populations using a rice dataset. The results show that the Pearson correlation method is both reliable and computationally efficient. In addition, we construct a genetic linkage map across generations utilizing the genetic distance calculated from the correlation converted recombination fractions. We observed map expansion, where the estimated genetic map length increases in later generations, reflecting improved resolution and detection of recombination events under finite marker density and sample size. This approach holds significant potential for broader applications in linkage mapping, quantitative trait loci (QTL) analysis, and design of breeding programs.

**Supplementary Information:**

The online version contains supplementary material available at 10.1007/s00122-026-05178-w.

## Introduction

Recombination fractions quantify the expected proportion of recombinant gametes produced between two genetic loci during meiosis, providing essential insights into the genetic architecture of organisms. Estimation of recombination fractions is a fundamental step in genetic research, playing a pivotal role in constructing linkage maps and understanding the inheritance patterns of crop genome in breeding populations. The estimation can be accomplished by certain mating designs, which enable structured and efficient data collection to facilitate the analysis of genetic linkages. Fast and accurate estimation of these recombination fractions enables researchers to infer linkage relationships among genetic markers, identify regions associated with specific traits, correct de novo genome assemblies, and facilitate marker-assisted selection and mating in plant and animal breeding programs (Collard and Mackill 2008; Fierst 2015; Varshney et al. 2005; Vidal et al. 2022). With the advancements in sequencing technologies, researchers can explore genetic architecture and trait associations more comprehensively. However, current methods often rely on specialized software and are computationally intensive, especially with large datasets (Humphreys et al. 2019). A fast and effective method for estimating recombination fractions is crucial for accelerating genomic research.

Recombination fractions are commonly estimated using the maximum likelihood (ML) method. The method for a backcross population is straightforward when the parental generation is homozygous, as is commonly achievable in plant systems, in which case the recombination fraction can be estimated by calculating the ratio of the recombinant gametes to the total number of gametes. The ML method for other mating designs such as $F_{2}$ and subsequent generations are more complicated due to the indistinguishable phases of the same observed genotypes. An iterative algorithm, such as expectation and maximization (EM) algorithm, have been developed to estimate the recombination fractions for $F_{2}$ populations, but can be computationally intensive when dealing with a large number of markers (Jiang and Zeng 1997; Xu 2012). Although recombination fraction estimation has been extensively studied in biparental populations, the direct use of Pearson correlation under idealized inbred $F_{t}$ settings has not been rigorously investigated. In particular, an explicit correlation‑based formulation for estimating recombination fractions in successive inbreeding generations is lacking, even though understanding recombination in these populations is important for capturing the increased resolution of genetic maps over successive generations. This highlights the need for a fast and straightforward method to estimate recombination fractions for $F_{t}$ populations.

The marker order of a genetic map is consistent with the order of the physical map when errors in constructing the physical map are well controlled. Advances in sequencing technology have enabled the development of accurate, high-quality physical maps, providing a reliable marker order (DeWan et al. 2002). While multi-point analysis can improve estimation when marker information is incomplete or marker order is unknown, in the fully informative setting with fixed marker order and no missing data, pairwise and multi-point analyses yield identical recombination fraction estimates for adjacent markers (Xu 2012). This makes pairwise recombination fraction estimation an efficient alternative for saving computational time and resources. However, most newly developed methods prioritize multi-point analysis with unknown marker orders (Meng et al. 2015). Some marker ordering algorithms, such as simulated annealing used in the software JoinMap, rely on pairwise recombination fraction for all markers (Stam 1993). Therefore, improving the speed of pairwise recombination fraction estimation is highly beneficial for genomic studies.

The current sequencing technology directly places markers in their correct order with marker distances naturally expressed as physical distances measured in base pairs (BP). However, physical distance and genetic distance do not exhibit a linear relationship, because genetic distance reflects recombination frequency rather than physical separation along the chromosome (Haldane 1919; Kosambi 2017). Genetic distance, typically measured in Morgans or centiMorgans (cM), is the quantity used in most genomic analyses. Therefore, the genetic distances between markers must be calculated before any applications of the genetic map to breeding. A typical application of genetic distances to breeding is genomic mating (as opposed to genomic selection). Matings between selected parents can be designed by breeders to produce progeny with the best performance (Akdemir and Sánchez 2016).This requires simulation of the genotypes of the progeny based on marker genotypes of the parents and the recombination fractions across markers.

In this research, we developed a new method using the Pearson correlation coefficient to estimate pairwise recombination fractions for all $F_{t}$ populations ($t \ge 2$). Our results demonstrated that the new method is consistent with the EM algorithm. We further applied the estimated recombination fractions to construct the genetic linkage maps for $F_{2}$, $F_{3}$ and $F_{4}$ populations that derived from the crosses of two *indica* rice varieties and observed map expansions as generations progress.

## Methods

### Relationship between correlation and recombination fraction

Let $A$ and $a$ be the two alleles of locus *A* in a biallelic system, where $aa$, $Aa$ and $AA$ are the three genotypes. Let *B* and *b* be the two alleles of locus *B*, where $bb$, $Bb$ and $BB$ are the three genotypes of locus *B*. Let $\theta$ be the recombination fraction between loci *A* and *B*. The value of $\theta$ ranges from 0 (complete linkage) to 0.5 (independent assortment). We use $X_{A}$ to indicate the number of *A* alleles in the genotype of locus *A* and $X_{B}$ the number of *B* alleles in the genotype of locus *B*, as shown below,

$$X_{A} = \left\{ {\begin{array}{*{20}c} 0 \\ 1 \\ 2 \\ \end{array} } \right.\begin{array}{*{20}c} {{\mathrm{for}}} \\ {{\mathrm{for}}} \\ {{\mathrm{for}}} \\ \end{array} \begin{array}{*{20}c} {aa} \\ {Aa} \\ {AA} \\ \end{array} {\text{ and }}X_{B} = \left\{ {\begin{array}{*{20}c} 0 \\ 1 \\ 2 \\ \end{array} } \right.\begin{array}{*{20}c} {{\mathrm{for}}} \\ {{\mathrm{for}}} \\ {{\mathrm{for}}} \\ \end{array} \begin{array}{*{20}c} {bb} \\ {Bb} \\ {BB} \\ \end{array}$$

Let $AB/AB$ and $ab/ab$ be the genotypes of the two inbred parents, and thus the genotype of their hybrid $F_{1}$ is $AB/ab$. The $F_{2}$ population is produced by self-fertilization of the $F_{1}$ individuals. The $F_{t}$ population refers to subsequent generations ($t > 2$) obtained through continued self-fertilization. A backcross population is generated by crossing $F_{t}$ individuals to one of the parental lines. For any $F_{t}$ generations, we found that the recombination fraction between *A* and *B* and the Pearson correlation coefficient $r$ between $X_{A}$ and $X_{B}$ have a simple relationship,

1 $$r = 1 - 2\theta$$

where $r$ is the Pearson correlation defined as

2 $$r = \frac{{{\mathrm{cov}} (X_{A} ,X_{B} )}}{{\sqrt {{\mathrm{var}} (X_{A} ){\mathrm{var}} (X_{B} )} }}$$

The relationship is valid under two assumptions: (1) no segregation distortion and (2) no crossover interference. Here, the Pearson correlation is induced by meiotic recombination in a controlled biparental population with inbred founders and should not be interpreted as linkage disequilibrium in a random mating population.

Estimating $\theta$ is straightforward using a backcross population. However, there is no simple formula to estimate $\theta$ in an $F_{2}$ population. The expectation and maximization (EM) algorithm is often used to estimate the recombination fraction. There is no formula for estimating the recombination fraction for $F_{t}$ populations for $t > 2$. The simple relationship between $\theta$ and $r$ allows us to estimated $\theta$ indirectly from $r$ using the reverse function,

3 $$\hat{\theta } = \frac{1}{2}(1 - \hat{r})$$

The estimation error is

4 $$s_{{\hat{\theta }}} = \sqrt {{\mathrm{var}} (\hat{\theta })} = \frac{1}{2}\sqrt {{\mathrm{var}} (\hat{r})} = \frac{{1 - r^{2} }}{{2\sqrt {n - 3} }}$$

This is due to the standard error of the Pearson correlation being (Fisher 1915)

$$s_{{\hat{r}}} = \sqrt {{\mathrm{var}} (\hat{r})} = \frac{{ \, 1 - r^{2} }}{{\sqrt {n - 3} }}$$

#### Proof of the relationship in a backcross (BC) population

Proof of the relationship in Eq. (1) for a BC population is very straightforward. Suppose that the BC population is derived from $AB/ab$ (*F*_1_) crossed back to $ab/ab$ (*P*_1_). The gametes from *P*_1_ are all *ab*. We only need to look at the gametes from *F*_1_. The four gametes and the joint distribution of $X_{A}$ and $X_{B}$ are given in Table 1, where $X_{A} = 0$ for $aa$, $X_{A} = 1$ for $Aa$, $X_{B} = 0$ for $bb$ and $X_{B} = 1$ for $Bb$. The four two-locus genotypes are also provided within the cells of the table. For example, the first cell is the combination of genotype *aa* from locus *A* and the genotype *bb* from locus *B*, and thus the two-locus genotype is *aabb* with a probability of $\tfrac{1}{2}(1 - \theta )$. This interpretation applies to the remaining three cells. The marginal expectation and variance of $X_{A}$ are ${\mathrm{E}}(X_{A} ) = 1/2$ and ${\mathrm{var}} (X_{A} ) = 1/4$, respectively. Similarly, the marginal expectation and variance of $X_{B}$ are ${\mathrm{E}}(X_{B} ) = 1/2$ and ${\mathrm{var}} (X_{B} ) = 1/4$, respectively. The covariance between $X_{A}$ and $X_{B}$ is

5 $${\mathrm{cov}} (X_{A} ,X_{B} ) = {\mathrm{E}}(X_{A} X_{B} ) - {\mathrm{E}}(X_{A} ){\mathrm{E}}(X_{B} ) = \frac{1}{4}(1 - 2\theta )$$

**Table 1:** Joint probability table of the four gametes in the backcross (BC) population

$X_{A} \backslash X_{{_{B} }}$	*bb* (0)	*Bb* (1)	Marginal
*aa* (0)	*aabb* $\tfrac{1}{2}(1 - \theta )$	*aaBb* $\tfrac{1}{2}\theta$	1/2
*Aa* (1)	*Aabb* $\tfrac{1}{2}\theta$	*AaBb* $\tfrac{1}{2}(1 - \theta )$	1/2
Marginal	1/2	1/2	1

The correlation coefficient is

6 $$r = \frac{{{\mathrm{cov}} (X_{A} ,X_{B} )}}{{\sqrt {{\mathrm{var}} (X_{A} ){\mathrm{var}} (X_{B} )} }} = \frac{{\tfrac{1}{4}(1 - 2\theta )}}{{\sqrt {\tfrac{1}{4} \times \tfrac{1}{4}} }} = 1 - 2\theta$$

Since we can directly observe the gametes in a BC population, there is no need for indirect estimation of the recombination fraction through correlation. One simply take the ratio of the number of recombinants to the total number of gametes (sample size) to estimate the recombination fraction, i.e.,

7 $$\hat{\theta } = \frac{{N_{Ab/ab} + N_{aB/ab} }}{{N_{AB/ab} + N_{Ab/ab} + N_{aB/ab} + N_{ab/ab} }}$$

where $N_{AB/ab}$, $N_{Ab/ab}$, $N_{aB/ab}$ and $N_{ab/ab}$ are the numbers of the gametes indicated by the subscripts.

#### Proof of the relationship in the ${{\boldsymbol{F}}}_{2}$ population

The joint probabilities for the two locus genotypes are summarized in Table 2. The expectation and variance of $X_{A}$ are ${\mathrm{E}}(X_{A} ) = 1$ and ${\mathrm{var}} (X_{A} ) = {\mathrm{E}}(X_{A}^{2} ) - {\mathrm{E}}^{2} (X_{A} ) = 1/2$, respectively. Similarly, the expectation and variance of $X_{B}$ are ${\mathrm{E}}(X_{B} ) = 1$ and ${\mathrm{var}} (X_{B} ) = {\mathrm{E}}(X_{B}^{2} ) - {\mathrm{E}}^{2} (X_{B} ) = 1/2$. The covariance between $X_{A}$ and $X_{B}$ is

8 $${\mathrm{cov}} (X_{A} ,X_{B} ) = {\mathrm{E}}(X_{A} X_{B} ) - {\mathrm{E}}(X_{A} ){\mathrm{E}}(X_{B} ) = \frac{1}{2}(1 - 2\theta )$$

**Table 2:** The joint probability table for two loci in the F_2_ population

$X_{A} \backslash X_{B}$	*bb* (0)	*Bb* (1)	*BB (*2)	Marginal
*aa* (0)	*aabb* $\tfrac{1}{4}(1 - \theta )^{2}$	*aaBb* $\tfrac{1}{2}\theta (1 - \theta )$	*aaBB* $\tfrac{1}{4}\theta^{2}$	$\tfrac{1}{4}$
*Aa* (1)	*Aabb* $\tfrac{1}{2}\theta (1 - \theta )$	*AaBb* $\tfrac{1}{2}\left[ {\theta^{2} + (1 - \theta )^{2} } \right]$	*AaBB* $\tfrac{1}{2}\theta (1 - \theta )$	$\tfrac{1}{2}$
*AA* (2)	*AAbb* $\tfrac{1}{4}\theta^{2}$	*AABb* $\tfrac{1}{2}\theta (1 - \theta )$	*AAbb* $\tfrac{1}{4}(1 - \theta )^{2}$	$\tfrac{1}{4}$
Marginal	$\tfrac{1}{4}$	$\tfrac{1}{2}$	$\tfrac{1}{4}$	1

Therefore, the correlation coefficient is

9 $$r = \frac{{{\mathrm{cov}} (X_{A} ,X_{B} )}}{{\sqrt {{\mathrm{var}} (X_{A} ){\mathrm{var}} (X_{B} )} }} = \frac{{\tfrac{1}{2}(1 - 2\theta )}}{{\sqrt {\tfrac{1}{2} \times \tfrac{1}{2}} }} = 1 - 2\theta$$

#### Proof of the relationship in an $F_{t}$ population for $t > 2$

Let $P_{t}$ be the frequency of genotype *aa*, $Q_{t} = P_{t}$ be the frequency of genotype *AA*, and $H_{t}$ be the frequency of genotype *Aa*. The corresponding genotype frequencies for locus *B* are the same because the situation is perfectly symmetrical. Heterozygosity at generation *t* is $H_{t} = \left( {1/2} \right)^{t - 1}$. The frequency of each homozygote is $P_{t} = Q_{t} = (1 - H_{t} )/2$. The joint probabilities for the two loci are summarized in Table 3, where the joint two locus genotypes have been ignored in the table, but they are the same as those shown in Table 2. The marginal expectation and variance are ${\mathrm{E}}(X_{A} ) = {\mathrm{E}}(X_{B} ) = 1$ and ${\mathrm{var}} (X_{A} ) = {\mathrm{var}} (X_{B} ) = 1 - H_{t}$, respectively. The covariance is ${\mathrm{cov}} (X_{A} ,X_{B} ) = (1 - H_{t} )(1 - 2\theta )$. The correlation coefficient is

10 $$r = \frac{{{\mathrm{cov}} (X_{A} ,X_{B} )}}{{\sqrt {{\mathrm{var}} (X_{A} ){\mathrm{var}} (X_{A} )} }} = \frac{{(1 - H_{t} )(1 - 2\theta )}}{{\sqrt {(1 - H_{t} )^{2} } }} = \frac{{(1 - H_{t} )(1 - 2\theta )}}{{(1 - H_{t} )}} = 1 - 2\theta$$

**Table 3:** The joint probability table of two loci in an $F_{t}$ population for $t \ge 2$

$X_{A} \backslash X_{B}$	*bb* (0)	*Bb* (1)	*BB* (2)	Marginal
*aa* (0)	$\tfrac{1}{2}(1 - H_{t} )(1 - \theta )^{2}$	$H_{t} \theta (1 - \theta )$	$\tfrac{1}{2}(1 - H_{t} )\theta^{2}$	$\tfrac{1}{2}(1 - H_{t} )$
*Aa* (1)	$H_{t} \theta (1 - \theta )$	$H_{t} \left[ {(1 - \theta )^{2} + \theta^{2} } \right]$	$H_{t} \theta (1 - \theta )$	$H_{t}$
*AA* (2)	$\tfrac{1}{2}(1 - H_{t} )\theta^{2}$	$(1 - H_{t} )\theta (1 - \theta )$	$\tfrac{1}{2}(1 - H_{t} )(1 - \theta )^{2}$	$\tfrac{1}{2}(1 - H_{t} )$
Marginal	$\tfrac{1}{2}(1 - H_{t} )$	$H_{t}$	$\tfrac{1}{2}(1 - H_{t} )$	1

### The EM algorithm for estimating recombination fraction

#### Conventional EM algorithm

We now introduce an intuitive EM algorithm for estimating the recombination fraction in the *F*_2_ population (Xu 2012) but extended to $F_{t}$ for $t \ge 2$. The $3 \times 3$ joint probability tables (Table 2 and Table 3) are used to construct the log likelihood function. Let $m_{ij}$ be the number of individuals from the *i*th row and the *j*th column of the joint probability table. Let $q_{ij}$ be the joint probability corresponding to the *i*th genotype of locus *A* and the *j*th genotype of locus *B* for $i,j = 1,2,3$. Among the $3 \times 3 = 9$ cells, the upper left cell (1,1) and lower right cell (3,3) represent individuals carrying no recombinants. The upper right cell (1,3) and the lower left cell (3,1) represent individuals carrying two recombinants. The four cells on the sides of the table, (1,2), (2,1), (2,3) and (3,2), represent individuals carrying one recombinant. The cell in the center, (2,2), is the double heterozygote (*AaBb*), who either carries two recombinants (*Ab/aB*) with probability $H_{t} \theta^{2}$ or non-recombinant (*AB/ab*) with probability $H_{t} (1 - \theta )^{2}$. Therefore, the log likelihood function is

11 $$\begin{aligned} L(\theta ) & = (m_{11} + m_{33} )\log (p_{11} + p_{33} ) + (m_{13} + m_{31} )\log (p_{13} + p_{31} ) \\ \quad + (m_{12} + m_{21} + m_{23} + m_{32} )\log (p_{12} + p_{21} + p_{23} + p_{32} ) \\ \quad + m_{22} \log (p_{22} ) \\ \end{aligned}$$

The mixture distribution for cell (2,2) makes the maximum likelihood solution complicated—no explicit solution is available. Let $n_{0}$ be the number of zero recombinants and $n_{2}$ be the number of double recombinants within cell (2,2). If we could separate the two classes in cell (2,2), the log likelihood function would be

12 $$\begin{aligned} L_{C} (\theta ) & = (m_{11} + m_{33} )\log (p_{11} + p_{33} ) + (m_{13} + m_{31} )\log (p_{13} + p_{31} ) \\ \quad + (m_{12} + m_{21} + m_{23} + m_{32} )\log (p_{12} + p_{21} + p_{23} + p_{32} ) \\ \quad + 2n_{0} \log (1 - \theta ) + 2n_{2} \log (\theta ) \\ \end{aligned}$$

The solution would be explicit, and no iterations would be required. Let us simplify Eq. (12) into

13 $$L_{C} (\theta ) = (m_{0} + 2n_{0} )\log (1 - \theta ) + (m_{2} + 2n_{2} )\log (\theta )$$

where

$$\begin{aligned} m_{0} = & 2(m_{11} + m_{33} ) + (m_{12} + m_{21} + m_{23} + m_{32} ) \\ m_{2} = & 2(m_{13} + m_{31} ) + (m_{12} + m_{21} + m_{23} + m_{32} ) \\ \end{aligned}$$

The derivative of the complete data log likelihood function with respect to the parameter is

14 $$\frac{\partial L(\theta )}{{\partial \theta }} = \frac{{m_{2} + 2n_{2} }}{\theta } - \frac{{m_{0} + 2n_{0} }}{1 - \theta }$$

Setting the derivative to zero and solving the equation lead to an explicit solution,

15 $$\hat{\theta } = \frac{{m_{2} + 2n_{2} }}{{(m_{2} + 2n_{2} ) + (m_{0} + 2n_{0} )}} = \frac{{m_{2} + 2n_{2} }}{{m_{2} + 2m_{22} + m_{0} }} = \frac{1}{2n}(m_{2} + 2n_{2} )$$

where $n$ is the sample size. The numerator represents the number of recombinant gametes, and the denominator represents the total number of gametes (twice the sample size). The EM algorithm simply replaces the missing values ($n_{2}$) by the posterior expectations,

16 $$\theta = \frac{1}{2n}\left[ {m_{2} + 2{\mathrm{E}}(n_{2} |\theta )} \right]$$

where

$${\mathrm{E}}(n_{2} |\theta ) = \rho m_{22}$$

is the posterior expectation of $n_{2}$ and

$$\rho = \frac{{\theta^{2} }}{{\theta^{2} + (1 - \theta )^{2} }}$$

is the posterior probability of double recombinants in cell (2,2). The EM iteration is rewritten as

17 $$\theta^{(t + 1)} = \frac{1}{2n}\left[ {m_{2} + 2{\mathrm{E}}(n_{2} |\theta^{(t)} )} \right] = \frac{1}{2n}(m_{2} + 2\rho^{(t)} m_{22} )$$

The variance of the EM estimate is obtained using Louis (1982) observed information,

18 $$I(\hat{\theta }) = - {\mathrm{E}}\left[ {\frac{{\partial^{2} L_{C} (\theta )}}{{\partial \theta^{2} }}} \right] - {\mathrm{var}} \left[ {\frac{{\partial L_{C} (\theta )}}{\partial \theta }} \right]$$

The variance of the EM estimate is

19 $${\mathrm{var}} (\hat{\theta }) = I^{ - 1} (\hat{\theta })$$

Derivation of Louis’ information is presented in Appendix 1 (Louis 1982).

#### Dempster’s EM algorithm

The expectation and maximization algorithm (Dempster et al. 1977) was originally developed for estimating recombination fraction in the *F*_2_ population, but the authors reformulated the estimation problem in a different way using the data from Rao’s book (Rao 1965). They defined alleles *A* and *B* as dominant over alleles *a* and *b* so that the investigators only observed four possible phenotypes, which are *A_B_*, *A_bb*, *aaB_* and *aabb*. In Rao’s book, these four phenotypes were simplified into *AB*, *Ab*, *aB* and *ab*, respectively. The joint probability table of the $3 \times 3 = 9$ genotypes and the 4 phenotypes are illustrated in Table 4 and Table 5, respectively. The probabilities of the four phenotypes from Table 5 are

$$\begin{aligned} \Pr (AB) = & \tfrac{1}{4}(1 - \theta )^{2} + \tfrac{1}{4}\theta (1 - \theta ) + \tfrac{1}{4}\theta (1 - \theta ) + \tfrac{1}{4}(1 - \theta )^{2} + \tfrac{1}{4}\theta (1 - \theta ) + \tfrac{1}{4}\theta^{2} + \tfrac{1}{4}\theta (1 - \theta ) + \tfrac{1}{4}\theta^{2} + \tfrac{1}{4}(1 - \theta )^{2} = \tfrac{1}{2} + \tfrac{1}{4}(1 - \theta )^{2} \\ \Pr (Ab) = & \tfrac{1}{4}\theta^{2} + \tfrac{1}{4}\theta (1 - \theta ) + \tfrac{1}{4}\theta (1 - \theta ) = \tfrac{1}{4}\left[ {(\theta^{2} + 2\theta (1 - \theta ) + (1 - \theta )^{2} - (1 - \theta )^{2} } \right] = \tfrac{1}{4}\left[ {1 - (1 - \theta )^{2} } \right] \\ \Pr (aB) = & \tfrac{1}{4}\theta^{2} + \tfrac{1}{4}\theta (1 - \theta ) + \tfrac{1}{4}\theta (1 - \theta ) = \tfrac{1}{4}\left[ {(\theta^{2} + 2\theta (1 - \theta ) + (1 - \theta )^{2} - (1 - \theta )^{2} } \right] = \tfrac{1}{4}\left[ {1 - (1 - \theta )^{2} } \right] \\ \Pr (ab) = & \tfrac{1}{4}(1 - \theta )^{2} \\ \end{aligned}$$

**Table 4:** The 16 possible genotypes of an ${\mathrm{F}}_{2}$ population from an F_1_ hybrid with genotype $AB/ab$ (the coupling phase)

Female\Male	*AB* $\tfrac{1}{2}(1 - \theta )$	*Ab* $\tfrac{1}{2}\theta$	*aB* $\tfrac{1}{2}\theta$	*ab* $\tfrac{1}{2}(1 - \theta )$
*AB* $\tfrac{1}{2}(1 - \theta )$*)*	*AB/AB* $\tfrac{1}{4}(1 - \theta )^{2}$	*AB/Ab* $\tfrac{1}{4}\theta (1 - \theta )$	*AB/aB* $\tfrac{1}{4}\theta (1 - \theta )$	*AB/ab* $\tfrac{1}{4}(1 - \theta )^{2}$
*Ab* $\tfrac{1}{2}\theta$	*Ab/AB* $\tfrac{1}{4}\theta (1 - \theta )$	*Ab/Ab* $\tfrac{1}{4}\theta^{2}$	*Ab/aB* $\tfrac{1}{4}\theta^{2}$	*Ab/ab* $\tfrac{1}{4}\theta (1 - \theta )$
*aB* $\tfrac{1}{2}\theta$	*aB/AB* $\tfrac{1}{4}\theta (1 - \theta )$	*aB/Ab* $\tfrac{1}{4}\theta^{2}$	*aB/aB* $\tfrac{1}{4}\theta^{2}$	*aB/ab* $\tfrac{1}{4}\theta (1 - \theta )$
*ab* $\tfrac{1}{2}(1 - \theta )$	*ab/AB* $\tfrac{1}{4}(1 - \theta )^{2}$	*ab/Ab* $\tfrac{1}{4}\theta (1 - \theta )$	*ab/aB* $\tfrac{1}{4}\theta (1 - \theta )$	*ab/ab* $\tfrac{1}{4}(1 - \theta )^{2}$

**Table 5:** The four possible phenotypes of an ${\mathrm{F}}_{2}$ population from an F_1_ hybrid with genotype $AB/ab$ (the coupling phase)

Female\Male	*AB* $\tfrac{1}{2}(1 - \theta )$	*Ab* $\tfrac{1}{2}\theta$	*aB* $\tfrac{1}{2}\theta$	*ab* $\tfrac{1}{2}(1 - \theta )$
*AB* $\tfrac{1}{2}(1 - \theta )$*)*	*AB* $\tfrac{1}{4}(1 - \theta )^{2}$	*AB* $\tfrac{1}{4}\theta (1 - \theta )$	*AB* $\tfrac{1}{4}\theta (1 - \theta )$	*AB* $\tfrac{1}{4}(1 - \theta )^{2}$
*Ab* $\tfrac{1}{2}\theta$	*AB* $\tfrac{1}{4}\theta (1 - \theta )$	*Ab* $\tfrac{1}{4}\theta^{2}$	*AB* $\tfrac{1}{4}\theta^{2}$	*Ab* $\tfrac{1}{4}\theta (1 - \theta )$
*aB* $\tfrac{1}{2}\theta$	*AB* $\tfrac{1}{4}\theta (1 - \theta )$	*AB* $\tfrac{1}{4}\theta^{2}$	*aB* $\tfrac{1}{4}\theta^{2}$	*aB* $\tfrac{1}{4}\theta (1 - \theta )$
*ab* $\tfrac{1}{2}(1 - \theta )$	*AB* $\tfrac{1}{4}(1 - \theta )^{2}$	*Ab* $\tfrac{1}{4}\theta (1 - \theta )$	*aB* $\tfrac{1}{4}\theta (1 - \theta )$	*ab* $\tfrac{1}{4}(1 - \theta )^{2}$

The authors transformed the parameter into $\pi = (1 - \theta )^{2}$ so that the probabilities of the four phenotypes are listed in Table 6. The log likelihood function for parameter $\pi$ is

20 $$L(\pi ) = n_{AB} \log (\tfrac{1}{2} + \tfrac{1}{4}\pi ) + (n_{Ab} + n_{aB} )\log (1 - \pi ) + n_{ab} \log \pi$$

**Table 6:** Frequencies of the four phenotypes in the original EM algorithm

Phenotype	Number of observations	Probability
*AB*	$n_{AB}$	$\tfrac{1}{2} + \tfrac{1}{4}\pi$
*Ab*	$n_{Ab}$	$\tfrac{1}{4}(1 - \pi )$
*aB*	$n_{aB}$	$\tfrac{1}{4}(1 - \pi )$
*ab*	$n_{ab}$	$\tfrac{1}{4}\pi$

If we decompose the first class of phenotype, *AB*, into two components, one is represented by $n_{0}$ with probability $1/2$ and the other is represented by $n_{2}$ with probability $\pi /4$, the log likelihood function would be

21 $$L_{C} (\pi ) = (n_{2} + n_{ab} )\log (\pi ) + (n_{Ab} + n_{aB} )\log (1 - \pi )$$

The solution would be

22 $$\pi = \frac{{n_{2} + n_{ab} }}{{n_{2} + n_{ab} + n_{Ab} + n_{aB} }}$$

Replacing $n_{2}$ by its posterior expectation yields

23 $$\pi = \frac{{{\mathrm{E}}(n_{2} |\pi ) + n_{ab} }}{{{\mathrm{E}}(n_{2} |\pi ) + n_{ab} + n_{Ab} + n_{aB} }}$$

where

24 $${\mathrm{E}}(n_{2} |\pi ) = \frac{{\tfrac{1}{4}\pi }}{{\tfrac{1}{2}{ + }\tfrac{1}{4}\pi }}n_{AB} = \rho n_{AB}$$

The EM iterative equation is

25 $$\pi^{(t + 1)} = \frac{{\pi^{(t)} n_{AB} + (2{ + }\pi^{(t)} )n_{ab} }}{{\pi^{(t)} n_{AB} + ({2 + }\pi^{(t)} )(n_{ab} + n_{Ab} + n_{aB} )}}$$

Once the iteration converges, we convert $\pi$ into $\theta$ using

26 $$\hat{\theta } = 1 - \sqrt {\hat{\pi }}$$

The variance of the EM estimate of $\pi$ is obtained via Louis (1982) observed information (see Appendix 1). Let us define the derivative by,

27 $$\nabla = \frac{\partial \theta }{{\partial \pi }} = - \frac{1}{{2\sqrt {\hat{\pi }} }}$$

The variance of the estimated recombination fraction is (Appendix 1)

28 $${\mathrm{var}} (\hat{\theta }) = \nabla {\mathrm{var}} (\hat{\pi })\nabla = \frac{1}{{4\hat{\pi }}}{\mathrm{var}} (\hat{\pi })$$

This is the so-called delta method.

### Examples for illustration

We extracted two pairs of loci of a rice genome as examples to demonstrate the differences between the new Pearson method and the two EM algorithms. The first pair of loci are Bin151 and Bin152 on chromosome 1 and the second pair of loci are Bin747 and Bin748 on chromosome 4. The observed counts ($m_{ij}$ for $i,j = 1,2,3$) are listed in Table 7. For example, the double heterozygote of the marker pair on Chr 1 is $m_{22} = 100$, and the count for the *AABB* genotype on Chr 4 is $m_{11} = 59$. Take the marker pair on Chr 1 as an example, the Pearson correlation coefficient is $\hat{r} = {0}{\mathrm{.94886}}$, which is converted into an estimated recombination fraction of $\hat{\theta } = (1 - \hat{r})/2 = (1 - 0.94886)/2 = 0.02557$. The standard error is

$$s_{{\hat{\theta }}} = \frac{{1 - \hat{r}^{2} }}{{2\sqrt {n - 3} }} = \frac{{1 - 0.94886^{2} }}{{2\sqrt {191 - 3} }} = 0.00363$$

**Table 7:** Counts of two locus genotypes of two marker pairs (Chr 1 and Chr 4)

Chr 1	BB	Bb	bb	Marginal
AA	41	2	0	43
Aa	3	100	0	103
aa	0	4	41	45
Marginal	44	106	41	191
Chr 4	BB	Bb	bb	Marginal
AA	59	2	2	63
Aa	4	103	3	110
aa	0	0	18	18
Marginal	63	105	23	191

The EM estimates from Dempster et al. (1977) dominance model and from (Xu 2012) codominance model along with the results obtained from the Newton Raphson ridge (NLPNRR) algorithm are given in Table 8. For the marker pair on Chr 1, the two EM algorithms generated slightly different estimates, both of which are lower than the Pearson correlation converted estimate. The standard error of the codominance model Xu (2012) is much smaller than that of the dominance model (Dempster et al. 1977), both higher than the standard error of the Pearson correlation converted recombination fraction.

**Table 8:** Estimated recombination fractions ($\hat{\theta }$) for two pairs of loci

Source	Method	Chr 1		Chr 4	
		Estimate	StdErr	Estimate	StdErr
Pearson	PCORR	0.02557	0.00363	0.05646	0.00777
Dominance	NLPNRR	0.02192	0.01093	0.03323	0.01486
Dominance	EM	0.02192	0.01093	0.03323	0.01486
Codominance	NLPNRR	0.02387	0.00792	0.03473	0.00957
Codominance	EM	0.02387	0.00791	0.03473	0.00956

PCORR, Pearson correlation; NLPNRR, Newton Raphson Ridge (a nonlinear program from SAS/IML); EM, EM algorithm

For the marker pair on Chr 4, the EM and NLPNRR estimates are very close to each other ($\hat{\theta } \approx 0.034$) but both are smaller than the Pearson correlation converted estimate ($\hat{\theta } = 0.05646$). The large deviation of the correlation converted recombination from the EM algorithm for this pair of markers is due to a greater segregation distortion. The theoretical proportions of the three genotypes are 0.25, 0.50 and 0.25, respectively. For the two markers on Chr 4, the Chi-square goodness of fit tests are $X_{2}^{2} = 25.607$ and $X_{2}^{2} = 18.6440$, respectively, corresponding to $$p = \begin{array}{*{20}l} {2.7507E - 6} \hfill \\ \end{array}$$ and $p = 0.0000894$ of the *p*-values. In contrast, the marker pair from Chr 1 show that the Chi-square tests are $X_{2}^{2} = 1.2199$ and $X_{2}^{2} = 2.4031$, respectively, corresponding to $p = 0.5434$ and $p = 0.3007$ of the *p*-values. This example indicates that the Pearson correlation method does not work well when there is segregation distortion.

## Results

### Simulation studies

**Fig. 1:**
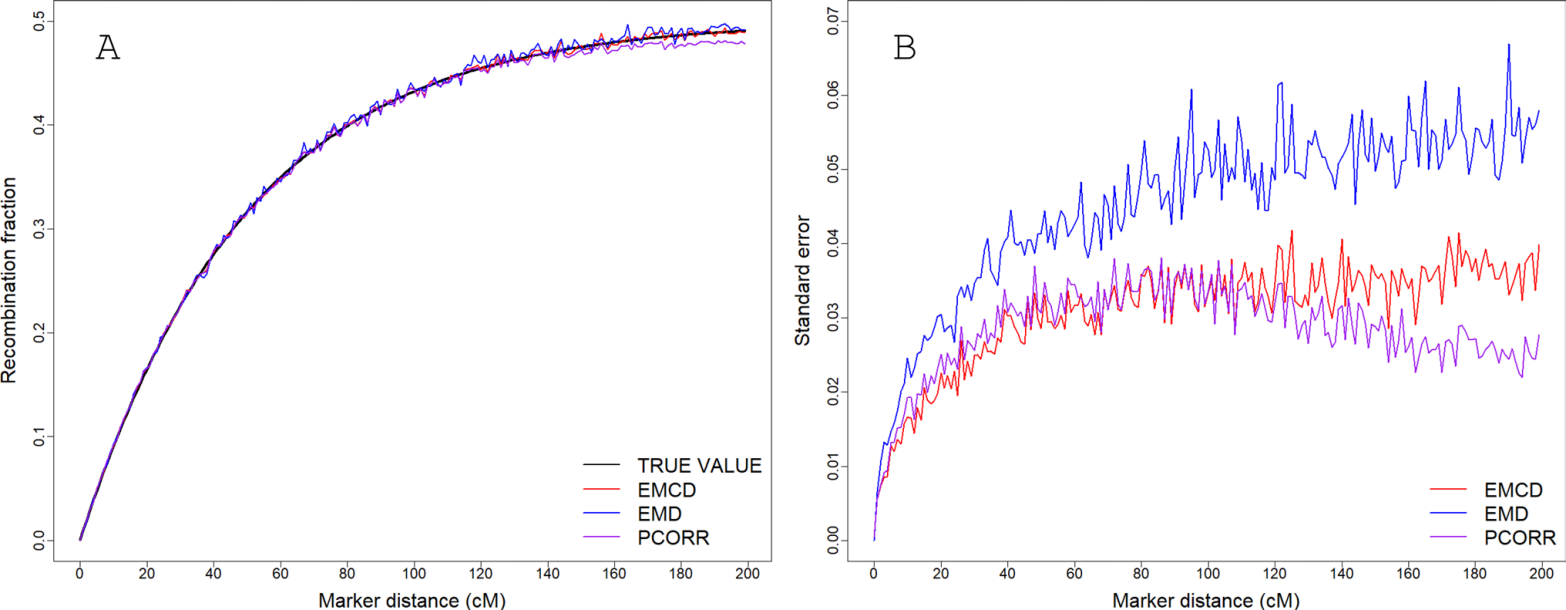
Averages (panel A) and standard errors (panel B) of 100 replicated simulations for estimated recombination fractions from three methods: (1) EM algorithm based on the codominance model (EMCD), (2) EM algorithm based on the dominance model (EMD) and (3) Pearson correlation converted method (PCORR)

We compared the results from the three methods for a simulated *F*_2_ population with $n = 200$ individuals. The simulations were replicated 100 times. Averages of the 100 replicates and the standard errors were evaluated for each of the three methods under each level of the marker distance ranging from $\theta = 0$ to $\theta = 0.5$ incremented by 0.01. The results are illustrated in Fig. 1. The three methods are the EM algorithm under the dominance model (EMD, Dempster et al 1977), the EM algorithm under the codominance (EMCD) model (Xu 2012) and the Pearson correlation converted estimate (PCORR). Figure 1 (panel A) compares the mean estimates of the three methods with the true recombination fractions. All three methods appear to be unbiased when the distance between the two markers are not too large. A slight downward bias was observed for the correlation method when the two markers are far away. Figure 1 (panel B) shows the standard errors of the estimated recombination fractions over the 100 replications for the three methods. Clearly, the EM algorithm under the dominance model (EMD) suffers the largest standard error across the whole range of the marker distance. The dominance model does not take advantage of the full information from the marker and thus bare the largest error. The EM codominance model (EMCD) and the Pearson correlation (PCORR) methods have similar standard errors when the recombination fraction is greater than 0.45, after which the standard error of the correlation method starts to decline Although the theoretical upper bound of the recombination fraction is 0.5, recombination fractions approaching this value are highly unlikely between adjacent markers in modern genomic studies with dense marker coverage.

To evaluate the robustness to departures from Mendelian segregation, we conducted simulations incorporating segregation distortion. Specifically, we re-weighted the baseline gamete (haplotype) probabilities implied by a given recombination fraction and then renormalized the probabilities to sum to one before sampling the gametes. Two representative distortion scenarios were considered: (i) allelic level distortion, where gametes carrying allele *A* were assigned higher weights (*AB* and *Ab*), and (ii) nonrecombinant distortion, where nonrecombinant gametes (*AB* and *ab*) were favored over recombinant gametes (*Ab* and *aB*).

Under the allelic level distortion, deviations in marginal genotype frequencies resulted in increased bias for the PCORR, slight bias for EMD and very little bias for EMCD (Figure S1). Under the nonrecombinant distortion, the estimated recombination fractions were systematically smaller than the nominal value used in the simulation, which is expected because favoring nonrecombinant gametes effectively reduces the transmitted recombinant frequency (Figure S2). The EMCD and the EMD methods were no better than the PCORR method in terms of bias. In this setting, the standard errors of PCORR and EMD were larger than those of EMCD.

### Rice populations (***F***_2_, ***F***_3_ and ***F***_4_)

The dataset comprises three successive generations of inbreeding, including 191 plants from each of the $F_{2}$, $F_{3}$ and $F_{4}$ generations developed through single-seed descent from a cross between two elite rice varieties, Zhenshan97 (ZS97) and Minghui63 (MH63). A total of 1696 marker bins were inferred and used as the genotypic data for analysis (Xu et al. 2024). Genotypes of the 1696 bins from the three generations along with the physical map of the 1696 markers are given in Supplementary Data S1. Summary statistics assessing the marker means and variances across generations are provided in Supplementary Table S1 to support the assumptions underlying our correlation-based method. We estimated the recombination fractions between adjacent markers using the Pearson correlation method, the EM dominance model (Dempster et al. 1977) and the EM codominance model (Xu 2012). The estimated recombination fractions were then converted into the additive distances (cM, centimorgan) using the Haldane mapping function (Haldane 1919),

29 $$\hat{d} = - \frac{1}{2}\log (1 - 2\hat{\theta }) \times 100$$

where $\hat{\theta }$ is the recombination fraction between two adjacent markers and $\hat{d}$ is the additive genetic distance (cM) between two adjacent markers.

### Comparisons between the EM algorithms and the Pearson correlation

**Fig. 2:**
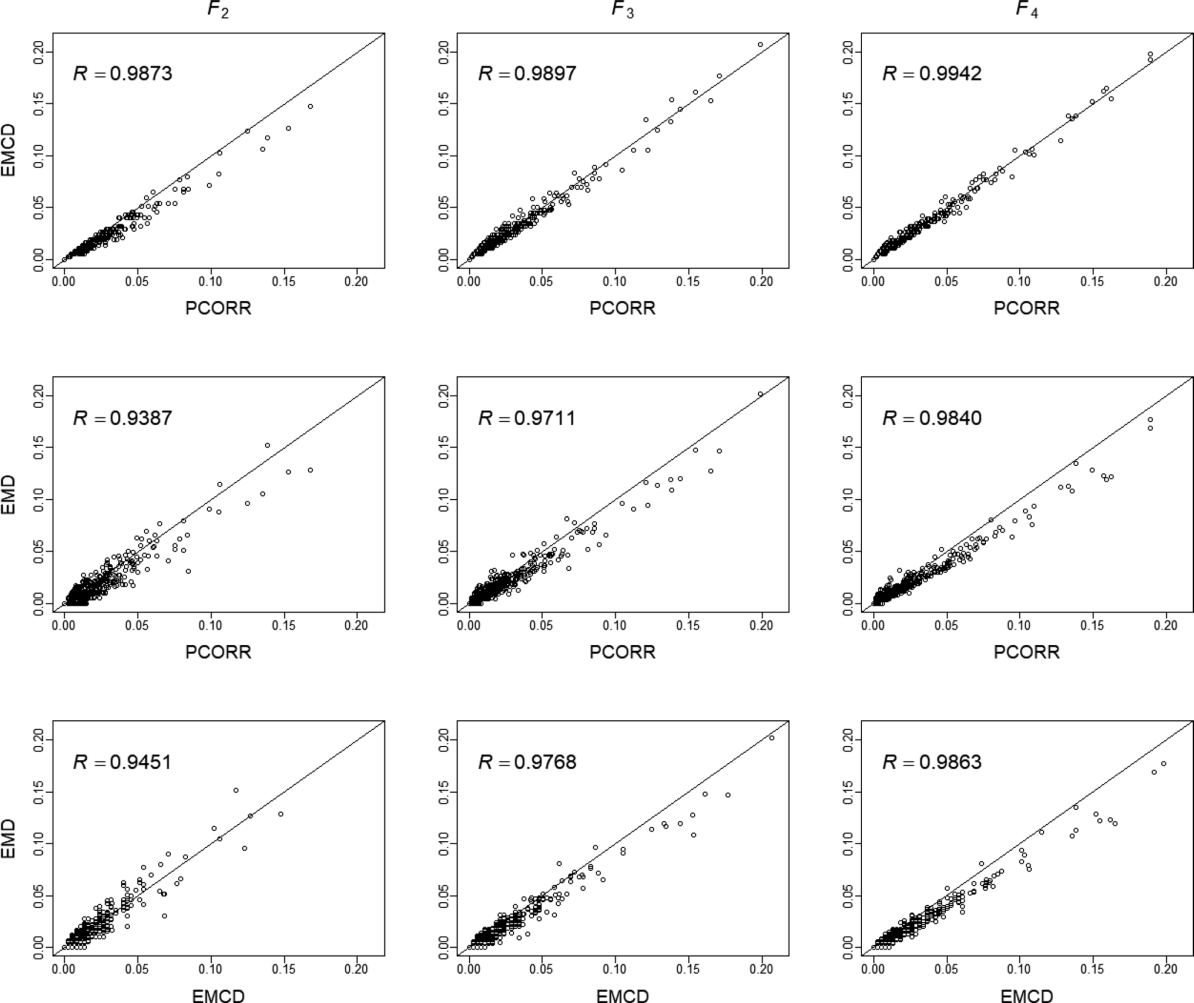
Comparison of recombination fractions estimated from three methods: the Pearson correlation method (PCORR), the EM algorithm under the dominance model (EMD) the EM algorithm under the codominance model (EMCD) across three generations (F_2_, F_3_ and F_4_)

The rice genome consists of 12 chromosomes and 1696 marker bins in this population. There are a total of 1684 recombination fractions between adjacent markers for each of the $F_{2}$, $F_{3}$ and $F_{4}$ populations. We compared the Pearson correlation converted recombination fractions and those estimated from the two EM algorithms (Fig. 2). The Pearson correlation method (PCORR) and the EM algorithms (EMD and EMCD) show strong consistency across all three generations. The correlation coefficients (*R*) between methods are higher as the generation number increases, with an average of about *R* = 0.96 for *F*_2_ to about *R* = 0.978 for *F*_3_ and about *R* = 0.988 for *F*_4_. Regarding the three methods, EMCD and PCORR have the highest correlation with an average value of greater than *R* = 0.988. The correlation between the EMD and PCORR method is slightly above *R* = 0.97. Likewise, the correlation between the two EM algorithms is above *R* = 0.97. Aside from accuracy, our method offers a major practical advantage in both simplicity and computational efficiency. PCORR completed the estimation across all marker pairs in just 0.2339 s. In contrast, EMD required 0.9598 s, and EMCD took 0.9988 s. This translates to a fourfold increase in speed for the Pearson method. The advantage becomes even more pronounced in high-dimensional datasets, where the non-iterative nature of the Pearson approach offers significant performance gains.

### Comparisons of linkage maps between methods and generations

**Fig. 3:**
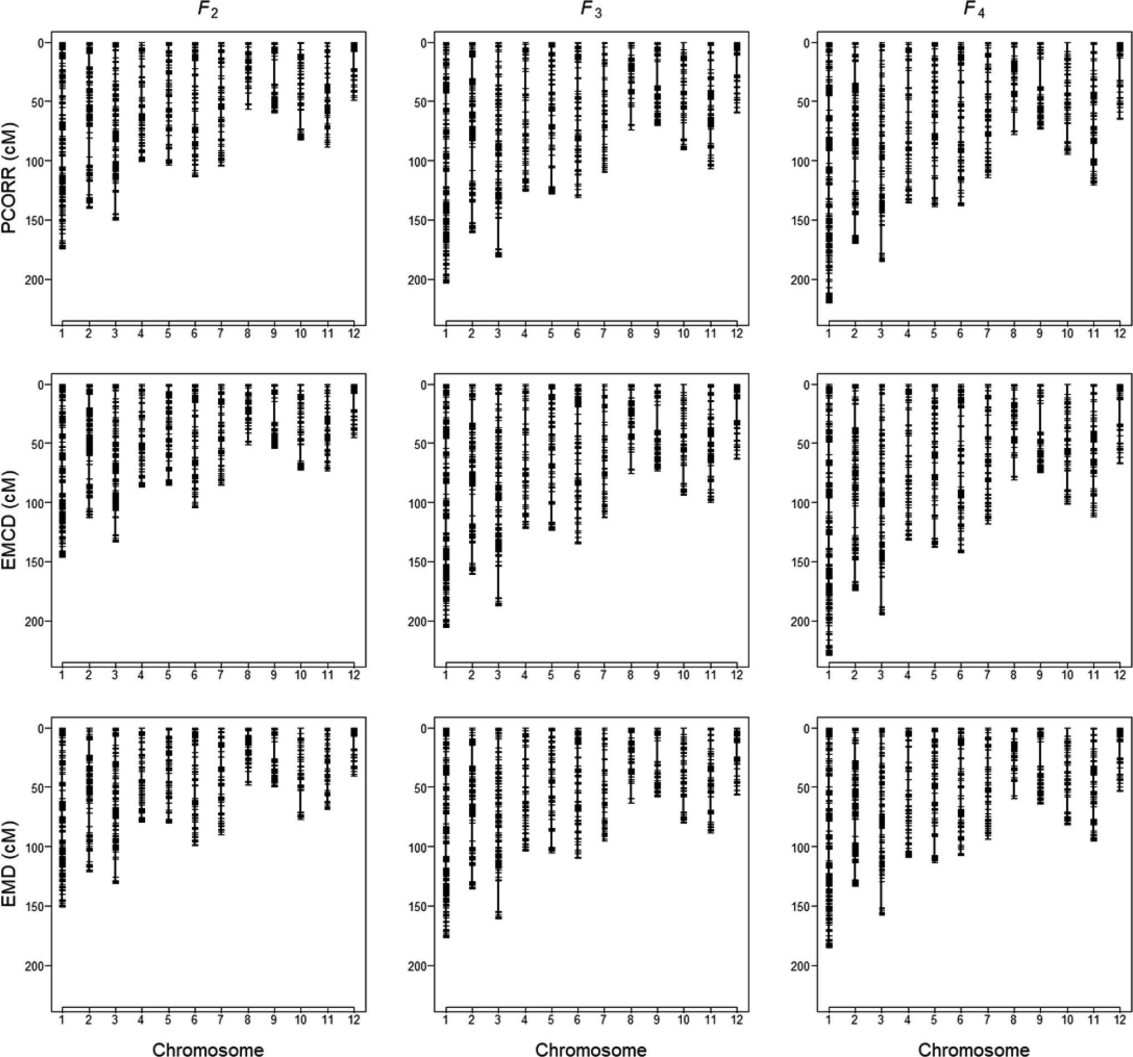
Comparison of linkage maps constructed from recombination fractions estimated from three methods: Pearson correlation (PCORR), EM algorithm under the dominance model (EMD) and EM algorithm under the codominance model (EMCD) across three generations (F_2_, F_3_ and F_4_)

From the estimated recombination fraction between adjacent markers, we constructed $3 \times 3 = 9$ linkage maps, one for each of the nine combinations of the 3 methods and the 3 generations (see Fig. 3). We observe a consistent increase in estimated map length from F_2_ to F_4_. This pattern is consistent with cumulative recombination events over successive generations. Because the comparison here is made across generations derived from the same genotyping platform, map inflation due to genotyping errors in high-density linkage maps (Lincoln & Lander 1992) is unlikely to apply here. Regarding the three methods, the EMD method produced the shortest maps compared to the maps produced with the PCORR and the EMCD methods. Maps produced by the PCORR and EMCD methods have similar lengths.

### Illustration of genomic mating

We used another simulated dataset to demonstrate how to use an estimated linkage map to facilitate genomic mating. It is well known that genomic data can be used to facilitate selection, called genomic selection (Meuwissen et al. 2001). If traits have a large component of dominance variation, we can use genomic data to facilitate genomic mating, i.e., from marker information of two potential parents to predict the genomic values of their progeny. Mating pairs with the greatest potential to produce high quality progeny will be chosen for reproduction. We simulated 500 individuals with 20 chromosomes and 100 cM in length of each chromosome covered by 101 evenly spaced markers (1 marker per cM). The entire genome was covered by 2020 biallelic markers evenly distributed along the genome. The population was initiated as an *F*_2_ population derived from two pure line breeds fixed for alternative alleles. Each marker was assigned an additive effect from a normal distribution $N(0,0.5)$ and a dominance effect from a normal distribution $N(0,1.0)$. The dominance effect represents the deviation of the heterozygote from the additive expectation, rather than complete dominance. The additive and dominance genotype codes of each marker are

$$Z_{jk} = \left\{ {\begin{array}{*{20}c} 0 \\ 1 \\ 2 \\ \end{array} } \right.\begin{array}{*{20}c} {} \\ {} \\ {} \\ \end{array} \begin{array}{*{20}c} {{\mathrm{for}}} \\ {{\mathrm{for}}} \\ {{\mathrm{for}}} \\ \end{array} \begin{array}{*{20}c} {aa} \\ {Aa} \\ {AA} \\ \end{array} {\text{ and }}W_{jk} = \left\{ {\begin{array}{*{20}c} 0 \\ 1 \\ 0 \\ \end{array} } \right.\begin{array}{*{20}c} {} \\ {} \\ {} \\ \end{array} \begin{array}{*{20}c} {{\mathrm{for}}} \\ {{\mathrm{for}}} \\ {{\mathrm{for}}} \\ \end{array} \begin{array}{*{20}c} {aa} \\ {Aa} \\ {AA} \\ \end{array}$$

respectively for $j = 1, \ldots ,n$ and $k = 1, \ldots ,m$, where $n = 500$ is the sample size and $m = 2020$ is the number of markers. The linear mixed model is

30 $$y = X\beta + Z\gamma + W\delta + \varepsilon$$

where $X\beta$ captures the fixed effects (population mean and systematic environmental effects), $\gamma$ is a vector of additive effects following a $\gamma \sim N(0,I\sigma_{\gamma }^{2} )$ distribution, $\delta$ is a vector of dominance effects following a $\delta \sim N(0,I\sigma_{\delta }^{2} )$ distribution. The expectation of *y* is ${\mathrm{E}}(y) = X\beta$ and the variance is

31 $${\mathrm{var}} (y) = V = ZZ^{T} \sigma_{\gamma }^{2} + WW^{T} \sigma_{\delta }^{2} + I\sigma_{\varepsilon }^{2}$$

Let us define the additive and dominance kinship matrices by

$$K_{\gamma } = \frac{1}{{C_{\gamma } }}ZZ^{T} {\text{and }}K_{\delta } = \frac{1}{{C_{\delta } }}WW^{T}$$

respectively, where $C_{\gamma } = {\mathrm{tr}}(ZZ^{T} )/n$ and $C_{\delta } = {\mathrm{tr}}(WW^{T} )/n$ are normalization factors for the two kinship matrices. The linear mixed model is then rewritten as

32 $$y = X\beta + \xi_{\gamma } + \xi_{\delta } + \varepsilon$$

where $\xi_{\gamma } = Z\gamma$ and $\xi_{\delta } = W\delta$. The variance of the linear mixed model becomes

33 $${\mathrm{var}} (y) = V = K_{\gamma } \sigma_{A}^{2} + K_{\delta } \sigma_{D}^{2} + I\sigma_{E}^{2}$$

where $\sigma_{A}^{2} = C_{\gamma } \sigma_{\gamma }^{2}$ is the additive variance and $\sigma_{D}^{2} = C_{\delta } \sigma_{\delta }^{2}$ is the dominance variance. The variance components are estimated using the restricted maximum likelihood (REML) method (Harville 1977). The genomic values of all individuals in the simulated population are obtained via the multivariate conditional expectations for the polygenic additive effects

34 $$\hat{\xi }_{\gamma } = \hat{\sigma }_{A}^{2} K_{\gamma } \hat{V}^{ - 1} (y - X\hat{\beta })$$

and for the polygenic dominance effects

35 $$\hat{\xi }_{\delta } = \hat{\sigma }_{D}^{2} K_{\delta } \hat{V}^{ - 1} (y - X\hat{\beta })$$

where

36 $$\hat{V} = K_{\gamma } \hat{\sigma }_{A}^{2} + K_{\delta } \hat{\sigma }_{D}^{2} + I\hat{\sigma }_{E}^{2}$$

The expected breeding values (EBV) of all individuals are

37 $$\hat{\xi } = \hat{\xi }_{\gamma } + \hat{\xi }_{\delta } = (\hat{\sigma }_{A}^{2} K_{\gamma } + \hat{\sigma }_{D}^{2} K_{\delta } )\hat{V}^{ - 1} (y - X\hat{\beta })$$

which are used to rank individuals for selection in descending or ascending order depending on the desired direction of selection. Selected individuals are going to the next stage—genomic mating. To predict the expected breeding value of a mating pair, i.e., the genomic value of the potential progeny of the mating pair, we need the estimated effects of all markers. These marker effects can be converted from the estimated additive and dominance polygenic effects using

38 $$\hat{\gamma } = Z^{T} (ZZ^{T} )^{ - 1} \hat{\xi }_{\gamma }$$

and

39 $$\hat{\delta } = W^{T} (WW^{T} )^{ - 1} \hat{\xi }_{\delta }$$

Derivations of these two equations are presented in Appendix 2.

Suppose that *s* sires and *d* dams are selected as the parents of the next generation. In conventional animal breeding programs, each sire is matted with *d*/*s* dams randomly. When genomic selection combined with genomic mating, the mating pairs are decided by the expected genomic values of their potential progeny. For example, let $s = 10$ and $d = 100$, where each sire is matted with $v = d/s = 100/10 = 10$ dams. In genomic mating, the mating pairs are decided by the expected genomic values of the progeny. Since we have fixed numbers of sires and dams and the allocation of dams to each sire is restricted to 10, there are a total of

40 $$N = \frac{d!}{{(v!)^{s} }} = \frac{100!}{{ \, (10!)^{10} }} = 2.357 \times 10^{92}$$

possible mating choices. Evaluating all the mating schemes is not possible and some heuristic search algorithm may be required. We now use a small example to illustrate the exhaustive search algorithm. Let $d = 6$ and $s = 2$ so that $v = d/s = 6/2 = 3$, leading to

41 $$N = \frac{d!}{{(v!)^{s} }} = \frac{6!}{{ \, (3!)^{2} }} = \frac{720}{{36}} = 20$$

possible mating schemes. This small example says that we select 6 female parents and 2 male parents for breeding. Each male is matted to 3 female parents. There are a total of 20 possible mating strategies. Table 9 lists all the 20 mating options. The dams listed in the first three columns are mated to sire 1 (S1) and the dams listed in the second three columns are mated to sire 2 (S2). For example, case 10 of the table shows that sire 1 is mated to dams 1, 2 and 5, while sire 2 is mated to dams 3, 4 and 6.

**Table 9:** Mating choices with two sires (S1 and S2) and six dams

Case	S1	S1	S1	S2	S2	S2
1	1	2	3	4	5	6
2	1	3	4	2	5	6
3	2	3	4	1	5	6
4	1	2	4	3	5	6
5	1	4	5	2	3	6
6	2	4	5	1	3	6
7	3	4	5	1	2	6
8	1	3	5	2	4	6
9	2	3	5	1	4	6
10	1	2	5	3	4	6
11	1	5	6	2	3	4
12	2	5	6	1	3	4
13	3	5	6	1	2	4
14	4	5	6	1	2	3
15	1	4	6	2	3	5
16	2	4	6	1	3	5
17	3	4	6	1	2	5
18	1	3	6	2	4	5
19	2	3	6	1	4	5
20	1	2	6	3	4	5

In the simulated population ($n = 500$), we selected $d = 9$ dams and $s = 3$ sires. The total number of mating choices is

$$N = \frac{9!}{{3!3!3!}} = \begin{array}{*{20}l} {1680} \hfill \\ \end{array}$$

Partial records of the 1680 mating choices are shown in Table 10. Females listed in the first three columns are mated with sire 1 (S1), females in the middle three columns are mated with sire 2, and females in the last three columns are mated with sire 3.

**Table 10:** Mating choices with three sires (S1, S2 and S3) and nine dams

Case	S1	S1	S1	S2	S2	S2	S3	S3	S3
1	1	2	3	4	5	6	7	8	9
2	1	2	3	4	5	7	6	8	9
3	1	2	3	4	5	8	6	7	9
4	1	2	3	4	5	9	6	7	8
5	1	2	3	4	6	7	5	8	9
6	1	2	3	4	6	8	5	7	9
7	1	2	3	4	6	9	5	7	8
8	1	2	3	4	7	8	5	6	9
9	1	2	3	4	7	9	5	6	8
10	1	2	3	4	8	9	5	6	7
1676	7	8	9	2	5	6	1	3	4
1677	7	8	9	3	4	5	1	2	6
1678	7	8	9	3	4	6	1	2	5
1679	7	8	9	3	5	6	1	2	4
1680	7	8	9	4	5	6	1	2	3

The simulated genotype data are presented in Supplementary Data S2, including the map (true map and estimated map) and the estimated additive and dominance effects of the markers. The simulated phenotypic values are presented in Supplementary Data S3. The additive and dominance kinship matrices calculated from genome-wide markers are presented in Supplementary Data S4. The 1680 mating choices are presented in Supplementary Data S5. We now describe the result of the genomic mating design. The estimated polygenic variances from the data are

$$\left[ {\begin{array}{*{20}c} {\hat{\sigma }_{A}^{2} } \\ {\hat{\sigma }_{D}^{2} } \\ {\hat{\sigma }_{E}^{2} } \\ \end{array} } \right] = \left[ {\begin{array}{*{20}r} \hfill {1571.939207} \\ \hfill {948.8769138} \\ \hfill {890.5598296} \\ \end{array} } \right]$$

**Fig. 4:**
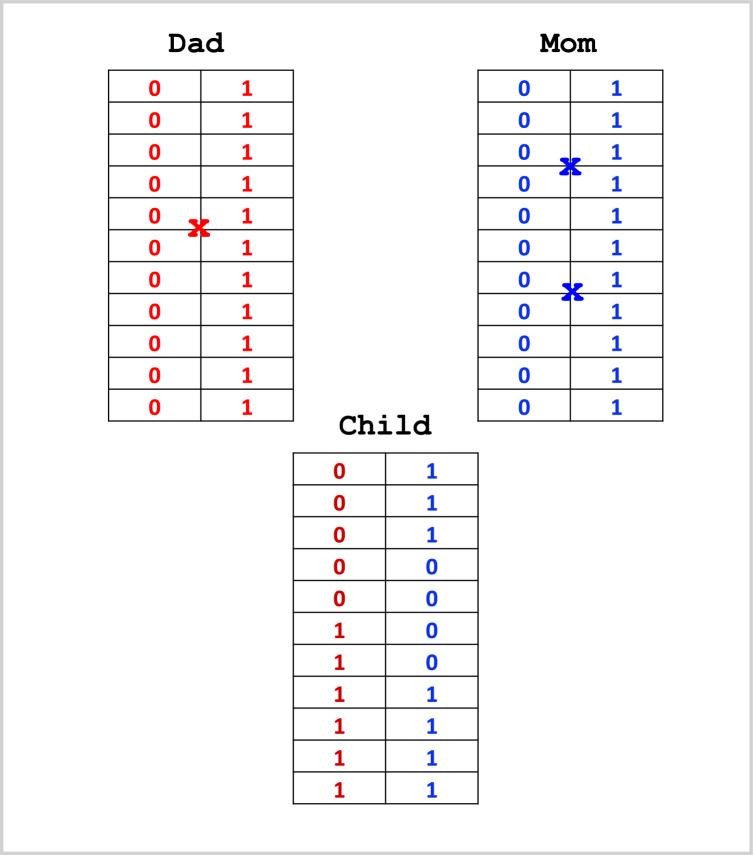
Simulation of two meiosis to generate the genotype of a progeny

**Fig. 5:**
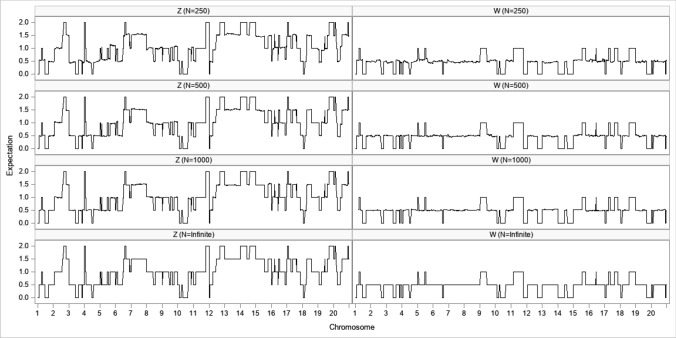
Comparison of the expected *Z* and *W* with the simulated *Z* and *W* for mating pair 415 (female) × 271 (male)

We sorted the 500 individuals based on their predicted polygenic effects (EBVs) in descending order. The top 12 individuals were selected, where the first 9 individuals were pretended to be the female parents (dams) and individuals ranked at 10, 11 and 12 were treated as the male parents (sires). The three male parents were then ranked separately and designated as sires 1, 2 and 3, respectively. The selected parents and the EBVs are listed in Table 11. There are two ways to generate the genotypes of a potential progeny from two parents, (1) expectation and (2) simulation. The expectation method is simple and straightforward. First, we generated the genotype probability table for the progeny from the genotypes of the two parents (Table 12). The expect genetic value for the progeny is

42 $$\hat{\xi } = {\mathrm{E}}(y) = \sum\limits_{k = 1}^{m} {{\mathrm{E}}(Z_{k} )\hat{\gamma }_{k} } + \sum\limits_{k = 1}^{m} {{\mathrm{E}}(W_{k} )\hat{\delta }_{k} }$$

where $m = 2020$ is the total number of markers. The simulation method requires more information from the parents: the linkage phases of the parental genotypes and the recombination fractions between all adjacent markers. This is why estimating the recombination fractions of markers is important in genomic mating. Although the averages of $Z_{k}$ and $W_{k}$ from a large number of repeated simulations will be very close to ${\mathrm{E}}(Z_{k} )$ and ${\mathrm{E}}(W_{k} )$, we can extract more information from the simulations, that is the standard errors of the expected breeding value, $s_{{\hat{\xi }}} = \sqrt {{\mathrm{var}} (y)}$. Figure 4 demonstrates the simulation process where we have one chromosome of length 100 cM covered by 11 evenly spaced markers. To simulate the gamete from the dad, we first sampled a Bernoulli variable with equal frequency, $\delta_{Sire} \sim {\mathrm{Bernouli}}(0.5)$, with 1 indicating the paternal allele (left) and 0 indicating the maternal allele (right) of the dad. In this example, $\delta_{Sire} = 1$, and thus the gamete started from the paternal allele of the dad. We then sampled a Poisson variable with a mean equal to 100 cM (1 Morgan), which is the length of the chromosome, $v_{Sire} \sim {\mathrm{Poisson}}(1)$. In this example, $v = 1$ and thus 1 crossover has happened. We then sampled a uniform variable between 0 and 100, $u\sim {\mathrm{Uniform}}(0,100)$, which determined the position of the crossover. In this example, $u = 45$ cM, and thus the crossover happened between the 5th and the 6th markers. The gamete from the dad has been formed (Fig. 4). To simulate the gamete from the mom, we simulated $\delta_{Dam} \sim {\mathrm{Bernouli}}(0.5)$ and $\delta_{Dam} = 0$ has happened, indicating that the gamete started with the maternal allele (right) of the mom. A Poisson variable was sampled from $v_{Dam} \sim {\mathrm{Poisson}}(1)$, and this time $v_{Dam} = 2$, meaning that two crossovers have taken place. We then simulated two uniform random numbers between 0 and 100. Assume that the two uniform random numbers happened to be 25 cM (between the 3rd and the 4th markers) and 63 cM (between the 7th and the 8th markers). The gamete carrying two crossovers from the mom then joined the gamete from the dad, forming the zygote of the child (Fig. 4). The simulation was repeated $N = 250$ times, $N = 500$ times and $N = 1000$ times. The expectation of $Z$ and $W$ were then substituted by the average of the *N* replicates,

$${\mathrm{E}}(Z_{k} ) \approx \frac{1}{N}\sum\limits_{j = 1}^{N} {Z_{jk} } {\text{ and E}}(W_{k} ) \approx \frac{1}{N}\sum\limits_{j = 1}^{N} {W_{jk} }$$

which were substituted to the expectations in Eq. (42) to evaluate the EBV of the mating pair. The expected genomic values of the mating strategy were supposed to be evaluated 1680 times, one for each of the sire and dam combinations. However, we only evaluated them once prior to searching for the best mating choice. There are $3 \times 9 = 27$ crosses of the 3 sires and the 9 dams, although in each mating strategy, only 9 of them are required. The 1680 mating choices only require different combinations of the 27 crosses. Using both the expectation method and the simulation method (*N* = 1000), we generated the $9 \times 3$ tables of the expected breeding values (EBVs) of the progeny (Table 13). We can see that the EBVs of the expectation method and the simulation method are almost identical. Supplementary Table S2 shows the standard errors from the simulation method, where the largest standard error is 18.5182 and the smallest standard error is 12.5469. This information is not available from the expectation method. For one particular pair of mating, the second dam and the third sire, the EBV is 73.8440 from the expectation method and 74.6620 from the simulation method (average of 1000 replicates). The difference is very small. Figure 5 compares ${\mathrm{E}}(Z)$ with $\overline{Z}$ and ${\mathrm{E}}(W)$ with $\overline{W}$ of the entire genome for this mating pair, where $\overline{Z}$ and $\overline{W}$ are the means of replicated simulations with $N = 250$, $N = 500$, and $N = 1000$, where $N = {\mathrm{Infinite}}$ represents the theoretical expectation. As the number of replicates increases, the average is closer to the expectation. In fact, $N = 250$ appears to be sufficient.

**Table 11:** Selected top 12 parents based on the highest EBV

Ranking	Dam	Sir	Additive (A)	Dominance (D)	EBV (A + D)
1	322		72.86057747	35.85302706	108.7136045
2	415		71.53942592	29.04470233	100.5841283
3	430		38.76231953	58.34962038	97.11193991
4	397		68.16370119	23.99620554	92.15990673
5	354		58.72335231	25.71762964	84.44098195
6	357		69.61596054	12.34829966	81.9642602
7	369		66.3747844	12.97791642	79.35270082
8	263		61.94307589	17.0379675	78.98104339
9	76		28.84427193	44.17245097	73.0167229
10		481	53.62989739	18.83248284	72.46238023
11		203	45.32493635	26.95508446	72.28002081
12		271	46.40929653	25.09100434	71.50030087

**Table 12:** Genotype probability table of progeny and the expectation of additive code and dominance code

Sire	Dam	aa (0)	Aa (1)	AA (2)	E(*Z*)	E(*W*)
aa	aa	1	0	0	0	0
aa	Aa	0.5	0.5	0	0.5	0.5
aa	AA	0	1	0	1	1
Aa	aa	0.5	0.5	0	0.5	0.5
Aa	Aa	0.25	0.5	0.25	1	0.5
Aa	AA	0	0.5	0.5	1.5	0.5
AA	aa	0	1	0	1	1
AA	Aa	0	0.5	0.5	1.5	0.5
AA	AA	0	0	1	2	0

**Table 13:** Expected breeding values (EBV) of 9 × 3 = 27 potential mating pairs

Female	Expectation			Simulation		
	Male 1	Male 2	Male 3	Male 1	Male 2	Male 3
1	58.0051	67.1442	50.7347	58.5843	67.3221	51.1481
2	59.5284	61.5243	73.8440	59.4423	62.1263	74.6620
3	57.0492	39.0092	37.0899	56.9004	38.7388	36.8073
4	64.5699	48.1993	52.8091	65.1212	48.3610	52.5051
5	45.0494	54.9185	55.9796	44.6055	55.5383	55.6576
6	68.3560	52.7611	51.3253	68.3559	52.5044	51.1482
7	59.7300	63.5272	49.8864	59.9604	62.9233	51.0129
8	62.9315	60.9344	46.0649	63.0743	61.7869	45.7872
9	41.1119	39.4849	27.8313	41.1946	38.6964	27.4801

We evaluated all the 1680 mating choices exhaustively and selected the top mating scheme, which is case 1190 in the permutations (see Supplementary Data S5). The results are summarized in Table 14, where the IDs and ranks of the 9 dams and 3 sires are presented followed by the EBVs and the corresponding standard errors from the simulations. The average EBV of the 9 mating pairs is $60.1251 \pm 16.4722$. The average EBV of all the 1689 potential mating choices is 53.6815. The gain of genomic mating over random mating is.

43 $$\Delta G = \frac{60.1251 - 53.6815}{{53.6815}} \times 100 = 12\%$$

**Table 14:** The optimal mating strategy among the 9 selected dams and 3 selected sires

Dam ID	Sire ID	Dam RNK	Sire RNK	EBV	STDERR
430	481	3	1	57.0492	16.2005
357	481	6	1	68.3560	14.9042
263	481	8	1	62.9315	14.8777
322	203	1	2	67.1442	15.9096
369	203	7	2	63.5272	16.0657
076	203	9	2	39.4849	16.9779
415	271	2	3	73.8440	16.7550
397	271	4	3	52.8091	18.5182
354	271	5	3	55.9796	18.0413

The average EBV of the 500 individuals (the whole sample) is 25.8107. The conventional genomic selection with random mating of the 3 sires and the 9 dams is 53.6815, which is already more than twice the average EBV of the entire population. The 12% gain is solely due to genomic mating in addition to genomic selection.

## Discussion

Recombination is a phenomenon that genetic material exchanges during meiosis, which is a mechanism to maintain genetic variation of populations. Estimating recombination fraction often requires a designed population from a cross between two lines in plants. In such populations, linkage disequilibrium between markers maintains the highest level and it is optimal to use such population to estimate recombination fractions between markers. We have demonstrated that Pearson correlation can be used as an alternative but simple method to estimate recombination fraction. In random mating populations linkage disequilibrium is influenced by evolutionary history and effective population size, rather than recombination frequency alone. Therefore, the Pearson correlation approach cannot be used for such a purpose. In human genetics, recombination fraction estimation therefore relies on alternative strategies, such as the identical-by-descent (IBD) approach (Gazal et al. 2017; Slatkin 2008; Zhou et al. 2020). If a population consists of many large families, we may consider using within-family correlation between markers to estimate recombination fractions because linkage disequilibrium will be maintained within-families. Within-family correlation is also called pooled correlation. Suppose that there are *m* families in a large random population, the within-family correlation may be estimated with

44 $$r_{XY} = \frac{{\sum\nolimits_{k = 1}^{m} {\left[ {\sum\nolimits_{j = 1}^{{n_{k} }} {(X_{jk} - \overline{X}_{k} )(Y_{jk} - \overline{Y}_{k} )} } \right]} }}{{\sqrt {\left[ {\sum\nolimits_{k = 1}^{m} {\sum\nolimits_{j = 1}^{{n_{k} }} {(X_{jk} - \overline{X}_{k} )^{2} } } } \right]\left[ {\sum\nolimits_{k = 1}^{m} {\sum\nolimits_{jk}^{{n_{k} }} {(Y_{jk} - \overline{Y}_{k} )^{2} } } } \right]} }}$$

where $X$ and $Y$ represent allelic numbers of genotypes for loci *X* and *Y*, respectively. Instead of denoting the two loci by loci *A* and *B*, we define the two loci by *X* and *Y* to avoid confusion caused by using *A* and *B* as subscripts.

The current sequencing technology provides both the SNP genotypes and the marker orders simultaneously. In standard GWAS and genomic selection frameworks, recombination fractions between adjacent markers are typically not modeled explicitly, with analyses instead relying directly on marker genotypes (Bush and Moore 2012; Misztal et al. 2021). Knowledge of recombination fractions between adjacent markers is needed in at least two situations: (1) simulation studies in genome research and (2) genomic mating. Simulations of genomic data require recombination fractions between adjacent markers. The recombination fractions are first converted into additive distances in Morgan or centiMorgan. The length of a simulated chromosome is then used to simulate the number of crossovers during meiosis. As demonstrated in the main text of the paper, simulation of gametogenesis requires accurate estimation of recombination fraction because that information determines the accuracy of the means of the genotype indicator variables, $\overline{Z}$ and $\overline{W}$, and the standard errors of the means.

One caveat of Pearson correlation approach to estimating the recombination fraction is when the correlation is negative. The equation to convert correlation into recombination fraction, Eq. (3), shows that if $r$ is negative, the converted recombination fraction is greater than 0.5, which is beyond the domain of this parameter. In such a situation, $r$ should be set to zero, leading to a converted recombination fraction of 0.5. The EM algorithm, however, does not have such a concern because the algorithm itself automatically restricts the solution within the legal boundaries. When the marker orders are fixed, recombination fractions are only estimated between adjacent markers, which are often small when the marker density is high. Under such conditions, negative correlations are unlikely to occur. However, they may arise in practice due to segregation distortion or stochastic sampling variability. For marker pairs that are far apart, estimation uncertainty is expected to increase for Pearson correlation-based estimators, as for other methods. However, in practical genetic map construction, recombination fraction estimates between distant markers play a limited role, as most algorithms primarily rely on local, adjacent-marker information for map construction.

Prior to applying the Pearson correlation approach, it is advisable to examine simple summary statistics to assess whether the underlying assumptions are satisfied. In particular, examining allele frequencies, marker means, and variances can serve as a useful diagnostic tool to assess whether the genotype distribution behaves as expected. As shown in Supplementary Table S1, the rice dataset closely matches theoretical expectations, especially in marker variances, supporting the use of the correlation-based estimator in this setting. In contrast, simulations with segregation distortion show that departures from Mendelian expectations can modify marginal genotype and haplotype frequencies, making the Pearson correlation approach unreliable in such settings. These findings underscore the necessity of reviewing basic summary statistics before using the Pearson correlation-based estimator.

It is very interesting that we have seen another example that a particular statistical method or algorithm was developed from a genetic data analysis problem. The original EM algorithm (Dempster et al. 1977) was developed using the very same genetic problem, estimation of recombination fraction. Back in 1977, genetic data were still scarce, and most genotype data were incomplete. The example data used by Dempster et al (1977) were perhaps from RFLP data, which were dominance markers. The EM estimation of recombination fraction from the dominance markers was subject to large error, and the comparison conducted in this study indeed reflected this fact. Genotypes from the current DNA sequence data are all codominant, but we still have missing values in the observed double heterozygote, which provides an opportunity for us to adopt the EM algorithm to estimate the recombination parameter. Other famous examples of scientists developing statistical methods motivated by genetics problems include the analysis of variance (ANOVA) developed by Fisher (1918) who partitioned the variance of human height into between-family variance and within-family variance (Fisher 1918). The between-family variance reflects part of the genetic variance of human height. Path analysis (Wright 1934) is another example of genetics problem being used to develop a statistical method. Wright (1934) developed the theory of path analysis as a new graphic theory to analyze pedigree data because he viewed pedigrees as graphs.

## Conflict of interest

The authors declare no conflict of interest.

## Electronic supplementary material

Below is the link to the electronic supplementary material.

Supplementary file1 (XLSX 3127 KB)

Supplementary file2 (XLSX 3304 KB)

Supplementary file3 (XLSX 21 KB)

Supplementary file4 (XLSX 5112 KB)

Supplementary file5 (XLSX 209 KB)

Supplementary file6 (PPTX 831 KB)

Supplementary file7 (DOCX 114 KB)

## Data Availability

The data generated and analyzed in this study are provided in the Supplementary Materials.

## Appendix 1

### Louis’ (1982) observed information for estimated recombination fraction

#### Conventional EM algorithm (the codominance model)

The complete data log likelihood function is

45 $$L_{C} (\theta ) = (m_{0} + 2n_{0} )\log (1 - \theta ) + (m_{2} + 2n_{2} )\log (\theta )$$

The derivative of the log likelihood function with respect to the parameter is

46 $$\frac{{\partial L_{C} (\theta )}}{\partial \theta } = \frac{{m_{2} + 2n_{2} }}{\theta } - \frac{{m_{0} + 2n_{0} }}{1 - \theta }$$

The second derivative (Hessian) is

47 $$H = \frac{{\partial^{2} L_{C} (\theta )}}{{\partial \theta^{2} }} = - \frac{{m_{2} + 2n_{2} }}{{\theta^{2} }} - \frac{{m_{0} + 2n_{0} }}{{(1 - \theta )^{2} }}$$

The expectation of the Hessian is

48 $${\mathrm{E}}(H) = {\mathrm{E}}\left[ {\frac{{\partial^{2} L_{C} (\theta )}}{{\partial \theta^{2} }}} \right] = - \frac{{m_{2} + 2{\mathrm{E}}(n_{2} |\theta )}}{{\theta^{2} }} - \frac{{m_{0} + 2{\mathrm{E}}(n_{0} |\theta )}}{{(1 - \theta )^{2} }} = - \frac{{m_{2} + 2\rho m_{22} }}{{\theta^{2} }} - \frac{{m_{0} + 2(1 - \rho )m_{22} }}{{(1 - \theta )^{2} }}$$

where

$$\rho = \frac{{\theta^{2} }}{{\theta^{2} + (1 - \theta )^{2} }},{\mathrm{E}}(n_{2} |\theta ) = \frac{{\theta^{2} }}{{\theta^{2} + (1 - \theta )^{2} }}m_{22} = \rho m_{22} ,$$

and

$${\mathrm{E}}(n_{0} |\theta ) = \frac{{(1 - \theta )^{2} }}{{\theta^{2} + (1 - \theta )^{2} }}m_{22} = (1 - \rho )m_{22}$$

The variance of the score (the first derivative) is

$$\begin{aligned} {\mathrm{var}} \left[ {\frac{{\partial L_{C} (\theta )}}{\partial \theta }} \right] = & {\mathrm{var}} \left[ {\frac{{m_{2} + 2n_{2} }}{\theta }} \right] + {\mathrm{var}} \left[ {\frac{{m_{0} + 2n_{0} }}{1 - \theta }} \right] - 2{\mathrm{cov}} \left[ {\frac{{m_{2} + 2n_{2} }}{\theta },\frac{{m_{0} + 2n_{0} }}{1 - \theta }} \right] \\ = & \frac{4}{{\theta^{2} }}{\mathrm{var}} (n_{2} |\theta ) + \frac{4}{{(1 - \theta )^{2} }}{\mathrm{var}} (n_{2} |\theta ) - \frac{8}{\theta (1 - \theta )}{\mathrm{cov}} (n_{0} ,n_{2} |\theta ) \\ = & \frac{4}{{\theta^{2} }}\rho (1 - \rho )m_{22} + \frac{4}{{(1 - \theta )^{2} }}\rho (1 - \rho )m_{22} + \frac{8}{\theta (1 - \theta )}\rho (1 - \rho )m_{22} \\ = & \left[ {\frac{4}{{\theta^{2} }} + \frac{4}{{(1 - \theta )^{2} }} + \frac{8}{\theta (1 - \theta )}} \right]\rho (1 - \rho )m_{22} \\ \end{aligned}$$

The information of the estimated parameter is

49 $$I(\theta ) = - {\mathrm{E}}\left[ {\frac{{\partial^{2} L_{C} (\theta )}}{{\partial \theta^{2} }}} \right] - {\mathrm{var}} \left[ {\frac{{\partial L_{C} (\theta )}}{\partial \theta }} \right]$$

#### EM algorithm under the dominance model

The log likelihood function for parameter $\pi$ is

50 $$L(\pi ) = n_{AB} \log (\tfrac{1}{2} + \tfrac{1}{4}\pi ) + (n_{Ab} + n_{aB} )\log (1 - \pi ) + n_{ab} \log \pi$$

If we decompose the first class of phenotype, *AB*, into two components, one is represented by $n_{0}$ with probability $1/2$ and the other is represented by $n_{2}$ with probability $\pi /4$, the complete data log likelihood function would bewhich is simplified into

$$\begin{aligned} L_{C} (\pi ) = & n_{0} \log (\tfrac{1}{2}) + n_{2} \log (\tfrac{1}{4}\pi ) + (n_{Ab} + n_{aB} )\log (1 - \pi ) + n_{ab} \log \pi \\ = & n_{0} \log (\tfrac{1}{2}) + n_{2} \log (\tfrac{1}{4}) + n_{2} \log (\pi ) + (n_{Ab} + n_{aB} )\log (1 - \pi ) + n_{ab} \log \pi \\ = & (n_{2} + n_{ab} )\log (\pi ) + (n_{Ab} + n_{aB} )\log (1 - \pi ) \\ \end{aligned}$$

51 $$L_{C} (\pi ) = (n_{2} + n_{ab} )\log (\pi ) + (n_{Ab} + n_{aB} )\log (1 - \pi )$$

The derivative with respect to the parameter is

$$\frac{{\partial L_{C} (\pi )}}{\partial \pi } = \frac{{n_{2} + n_{ab} }}{\pi } - \frac{{n_{Ab} + n_{aB} }}{1 - \pi }$$

The second derivative is

52 $$\frac{{\partial^{2} L_{C} (\pi )}}{{\partial \pi^{2} }} = - \frac{{n_{2} + n_{ab} }}{{\pi^{2} }} - \frac{{n_{Ab} + n_{aB} }}{{(1 - \pi )^{2} }}$$

The expectation is

$${\mathrm{E}}\left[ {\frac{{\partial^{2} L_{C} (\pi )}}{{\partial \pi^{2} }}} \right] = - \frac{{{\mathrm{E}}(n_{2} |\pi ) + n_{ab} }}{{\pi^{2} }} - \frac{{n_{Ab} + n_{aB} }}{{(1 - \pi )^{2} }} = - \frac{{\rho n_{AB} + n_{ab} }}{{\pi^{2} }} - \frac{{n_{Ab} + n_{aB} }}{{(1 - \pi )^{2} }}$$

where

$$\rho = \frac{{\tfrac{1}{4}\pi }}{{\tfrac{1}{2}{ + }\tfrac{1}{4}\pi }} = \frac{\pi }{{2{ + }\pi }}$$

and

$${\mathrm{E}}(n_{2} |\pi ) = \frac{\pi }{{2{ + }\pi }}n_{AB} = \rho n_{AB}$$

The variance of the score is

53 $${\mathrm{var}} \left[ {\frac{{\partial L_{C} (\pi )}}{\partial \pi }} \right] = \frac{1}{{\pi^{2} }}{\mathrm{var}} (n_{2} |\pi ) = \frac{1}{{\pi^{2} }}\rho (1 - \rho )n_{AB}$$

The information of the estimated $\pi$ s

54 $$\begin{aligned} I(\hat{\pi }) = & - E\left[ {\frac{{\partial^{2} L_{C} (\pi )}}{{\partial \pi^{2} }}} \right] - {\mathrm{var}} \left[ {\frac{{\partial L_{C} (\pi )}}{\partial \pi }} \right] \\ = & \frac{{\rho n_{AB} + n_{ab} }}{{\pi^{2} }} + \frac{{n_{Ab} + n_{aB} }}{{(1 - \pi )^{2} }} - \frac{{\rho (1 - \rho )n_{AB} }}{{\pi^{2} }} \\ = & \frac{{n_{ab} + \rho^{2} n_{AB} }}{{\pi^{2} }} + \frac{{n_{Ab} + n_{aB} }}{{(1 - \pi )^{2} }} \\ = & \frac{{(n_{ab} + \rho^{2} n_{AB} )(1 - \pi )^{2} }}{{\pi^{2} (1 - \pi )^{2} }} + \frac{{(n_{Ab} + n_{aB} )\pi^{2} }}{{\pi^{2} (1 - \pi )^{2} }} \\ = & \frac{{(n_{ab} + \rho^{2} n_{AB} )(1 - \pi )^{2} + (n_{Ab} + n_{aB} )\pi^{2} }}{{\pi^{2} (1 - \pi )^{2} }} \\ \end{aligned}$$

The variance is

55 $${\mathrm{var}} (\hat{\pi }) = I^{ - 1} (\hat{\pi }) = = \frac{{\pi^{2} (1 - \pi )^{2} }}{{(n_{ab} + \rho^{2} n_{AB} )(1 - \pi )^{2} + (n_{Ab} + n_{aB} )\pi^{2} }}$$

The recombination fraction is

56 $$\hat{\theta } = 1 - \sqrt {\hat{\pi }}$$

The variance of the recombination fraction is

57 $${\mathrm{var}} (\hat{\theta }) = \Delta {\mathrm{var}} (\hat{\pi })\Delta = \left[ { - \frac{1}{{2\sqrt {\hat{\pi }} }}} \right]{\mathrm{var}} (\hat{\pi })\left[ { - \frac{1}{{2\sqrt {\hat{\pi }} }}} \right] = \frac{1}{{4\hat{\pi }}}{\mathrm{var}} (\hat{\pi })$$

where

$$\Delta = - \frac{1}{{2\sqrt {\hat{\pi }} }}$$

The standard error is

58 $$s_{{\hat{\theta }}} = \sqrt {\frac{1}{{4\hat{\pi }}}{\mathrm{var}} (\hat{\pi })} = \frac{1}{{2\sqrt {\hat{\pi }} }}\sqrt {{\mathrm{var}} (\hat{\pi })}$$

## Appendix 2

### Relationship between marker effects and polygenic effects

Marker effects can be estimated in several different ways, but the easiest way is to convert them from the already estimated polygenic effects, as shown below,

59 $$\hat{\gamma } = Z^{T} (ZZ^{T} )^{ - 1} \hat{\xi }_{\gamma }$$

and

60 $$\hat{\delta } = W^{T} (WW^{T} )^{ - 1} \hat{\xi }_{\delta }$$

We can derive these two equations using the conditional expectation theorem of multivariate normal distribution. Let us define a vector with three components, $y$, $\gamma$ and $\delta$. The expectations and variance of this vector are

61 $${\mathrm{E}}\left[ {\begin{array}{*{20}c} y \\ \gamma \\ \delta \\ \end{array} } \right] = \left[ {\begin{array}{*{20}c} {X\beta } \\ 0 \\ 0 \\ \end{array} } \right]$$

and

62 $${\mathrm{var}}\left[ {\begin{array}{*{20}c} y \\ \gamma \\ \delta \\ \end{array} } \right] = \left[ {\begin{array}{*{20}c} V & {Z\sigma_{\gamma }^{2} } & {W\sigma_{\delta }^{2} } \\ {Z^{T} \sigma_{\gamma }^{2} } & {I\sigma_{\gamma }^{2} } & 0 \\ {W^{T} \sigma_{\delta }^{2} } & 0 & {I\sigma_{\delta }^{2} } \\ \end{array} } \right]\left[ {\begin{array}{*{20}c} {X\beta } \\ 0 \\ 0 \\ \end{array} } \right]$$

where

$$V = {\mathrm{var}} (y) = K_{\gamma } \sigma_{A}^{2} + K_{\delta } \sigma_{D}^{2} + I\sigma_{E}^{2}$$

The conditional expectation of $\gamma$ given $y$ is

63 $$\hat{\gamma } = {\mathrm{E}}(\gamma |y) = {\mathrm{cov}} (\gamma ,y){\mathrm{var}} (y)^{ - 1} \left[ {y - {\mathrm{E}}(y)} \right] = Z^{T} \sigma_{\gamma }^{2} V^{ - 1} (y - X\beta )$$

where $\sigma_{A}^{2} = C_{\gamma } \sigma_{\gamma }^{2}$ and thus

64 $$\hat{\gamma } = {\mathrm{E}}(\gamma |y) = \frac{1}{{C_{\gamma } }}Z^{T} \sigma_{A}^{2} V^{ - 1} (y - X\beta )$$

The conditional expectation of $\delta$ given $y$ is

65 $$\hat{\delta } = {\mathrm{E}}(\delta |y) = {\mathrm{cov}} (\delta ,y){\mathrm{var}} (y)^{ - 1} \left[ {y - {\mathrm{E}}(y)} \right] = W^{T} \sigma_{\delta }^{2} V^{ - 1} (y - X\beta )$$

where $\sigma_{D}^{2} = C_{\delta } \sigma_{\delta }^{2}$ and thus

66 $$\hat{\delta } = {\mathrm{E}}(\delta y) = \frac{1}{{C_{\delta } }}W^{T} \sigma_{D}^{2} V^{ - 1} (y - X\beta )$$

The polygenic additive effects are

67 $$\hat{\xi }_{\gamma } = \sigma_{A}^{2} K_{\gamma } V^{ - 1} (y - X\beta )$$

Substituting this into Eq. (59) leads to

68 $$\begin{aligned} \hat{\gamma } = & Z^{T} (ZZ^{T} )^{ - 1} \hat{\xi }_{\gamma } \\ = & Z^{T} (ZZ^{T} )^{ - 1} K_{\gamma } \sigma_{A}^{2} (K_{\gamma } \sigma_{A}^{2} + K_{\delta } \sigma_{D}^{2} )(y - X\hat{\beta }) \\ = & \frac{1}{{C_{\gamma } }}Z^{T} \sigma_{A}^{2} (K_{\gamma } \sigma_{A}^{2} + K_{\delta } \sigma_{D}^{2} )(y - X\hat{\beta }) \\ \end{aligned}$$

because $K_{\gamma } = ZZ^{T} /C_{\gamma }$. Similarly, the polygenic dominance effects are

69 $$\hat{\xi }_{\delta } = \sigma_{D}^{2} K_{\delta } V^{ - 1} (y - X\beta )$$

Substituting this into Eq. (60) leads to

70 $$\begin{aligned} \hat{\delta } = & W^{T} (WW^{T} )^{ - 1} \hat{\xi }_{\delta } \\ = & W^{T} (WW^{T} )^{ - 1} K_{\delta } \sigma_{D}^{2} (K_{\gamma } \sigma_{A}^{2} + K_{\delta } \sigma_{D}^{2} )(y - X\hat{\beta }) \\ = & \frac{1}{{C_{\delta } }}W^{T} \sigma_{D}^{2} (K_{\gamma } \sigma_{A}^{2} + K_{\delta } \sigma_{D}^{2} )(y - X\hat{\beta }) \\ \end{aligned}$$

because $$K_{\delta } = WW^{T} /C_{\delta }$$.
