# Supplementary material for: Estimating recombination fraction via Pearson correlation
Source: Theor Appl Genet. 2026 Feb 14;139(3):70. doi: 10.1007/s00122-026-05178-w (PMC12906585; doi:10.1007/s00122-026-05178-w)
Supplement: Supplementary file 6 — Supplementary file6 (PPTX 831 KB) [file 122_2026_5178_MOESM6_ESM.pptx]

## Slide 1
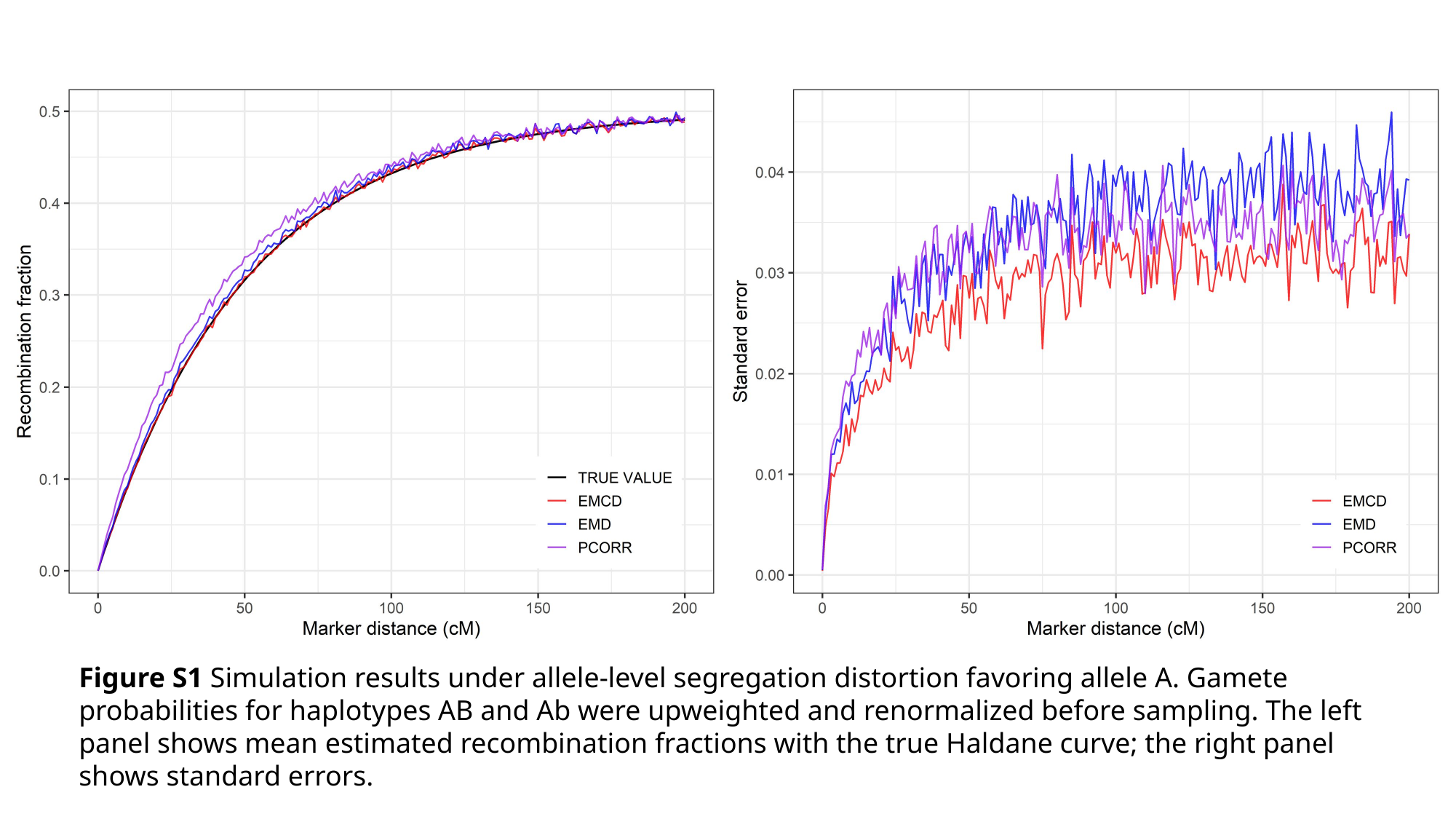

Figure S1 Simulation results under allele-level segregation distortion favoring allele A. Gamete probabilities for haplotypes AB and Ab were upweighted and renormalized before sampling. The left panel shows mean estimated recombination fractions with the true Haldane curve; the right panel shows standard errors.

## Slide 2
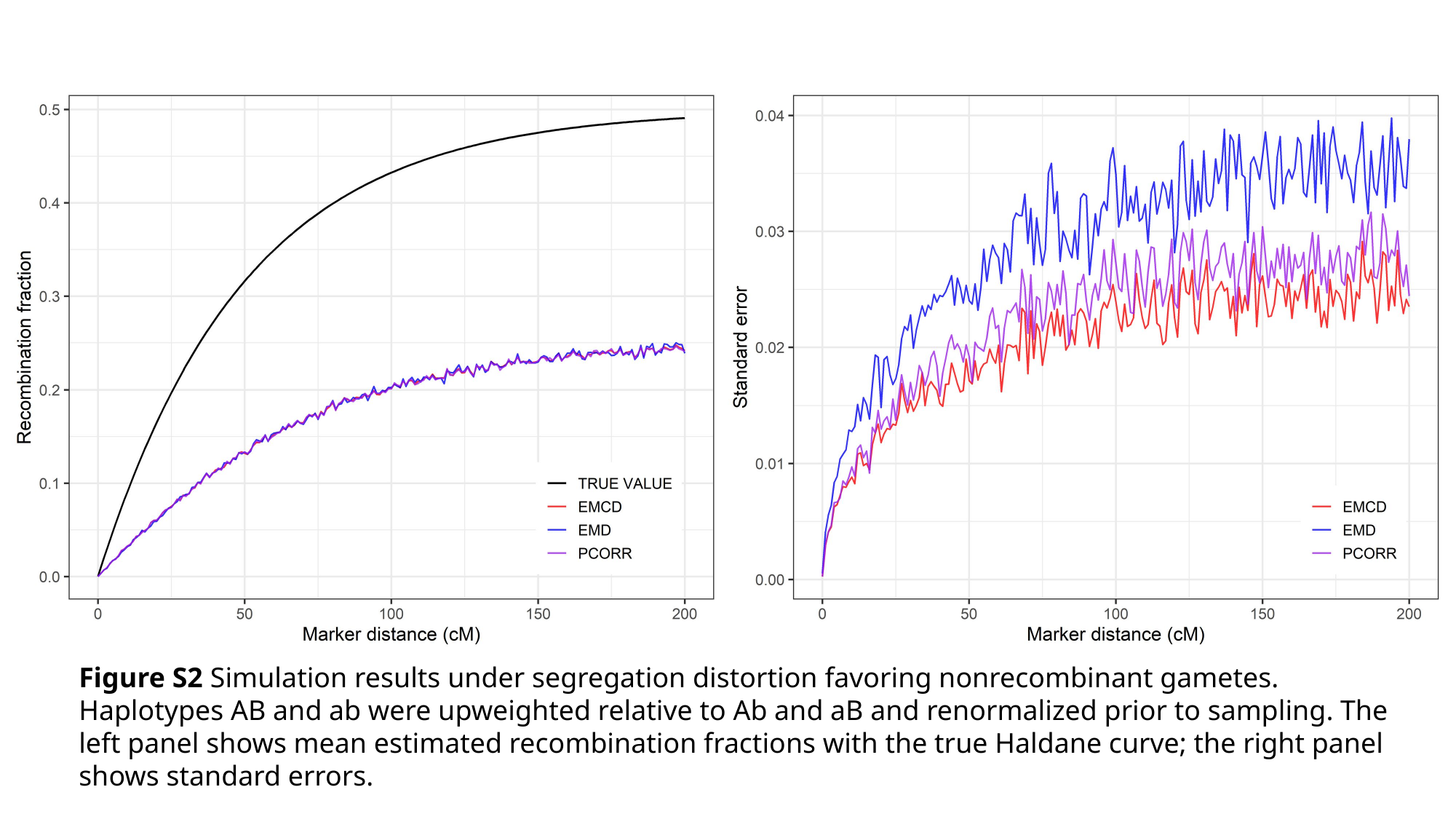

Figure S2 Simulation results under segregation distortion favoring nonrecombinant gametes. Haplotypes AB and ab were upweighted relative to Ab and aB and renormalized prior to sampling. The left panel shows mean estimated recombination fractions with the true Haldane curve; the right panel shows standard errors.
