# Supplementary material for: Estimating recombination fraction via Pearson correlation
Source: Theor Appl Genet. 2026 Feb 14;139(3):70. doi: 10.1007/s00122-026-05178-w (PMC12906585; doi:10.1007/s00122-026-05178-w)
Supplement: Supplementary file 7 — Supplementary file7 (DOCX 114 KB) [file 122_2026_5178_MOESM7_ESM.docx]

**Supplementary Table S1** Comparison between theoretical and observed values for mean and variance across populations using bias and Mean Square Error (MSE). The theoretical value for each population is shown in parentheses next to the bias values.


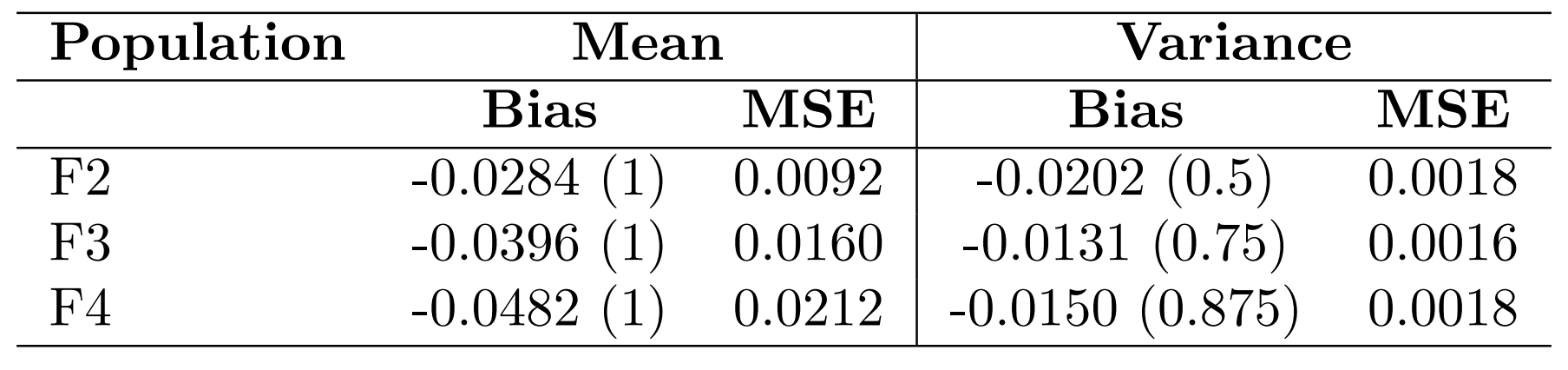


**Supplementary Table S2** Standard errors of EBVs of 9×3 = 27 potential mating pairs

| Female | S1 | S2 | S3 |
| --- | --- | --- | --- |
| 1 | 13.7844 | 15.9096 | 15.4766 |
| 2 | 12.5469 | 14.2206 | 16.7550 |
| 3 | 16.2005 | 16.7637 | 18.4444 |
| 4 | 16.7824 | 17.2390 | 18.5182 |
| 5 | 16.3566 | 14.6287 | 18.0413 |
| 6 | 14.9042 | 15.4776 | 17.5317 |
| 7 | 15.8693 | 16.0657 | 18.2786 |
| 8 | 14.8777 | 16.3857 | 17.7839 |
| 9 | 14.7447 | 16.9779 | 16.9490 |
